# Potentially Beneficial Effects on Healthy Aging by Supplementation of the EPA-Rich Microalgae *Phaeodactylum tricornutum* or Its Supernatant—A Randomized Controlled Pilot Trial in Elderly Individuals

**DOI:** 10.3390/md20110716

**Published:** 2022-11-15

**Authors:** Lena Stiefvatter, Konstantin Frick, Katja Lehnert, Walter Vetter, Alexander Montoya-Arroyo, Jan Frank, Ulrike Schmid-Staiger, Stephan C. Bischoff

**Affiliations:** 1 Institute of Clinical Nutrition, University of Hohenheim, Fruwirthstr. 12, 70593 Stuttgart, Germany; 2 Fraunhofer Institute for Interfacial Engineering and Biotechnology, 70569 Stuttgart, Germany; 3 Institute of Food Chemistry, University of Hohenheim, 70593 Stuttgart, Germany; 4 Department of Food Biofunctionality, Institute of Nutritional Sciences, University of Hohenheim, 70593 Stuttgart, Germany

**Keywords:** elderly, inflammageing, *Phaeodactylum tricornutum*, omega-3-fatty acids, eicosapentaenoic acid, fucoxanthin, β-glucan, chrysolaminarin

## Abstract

Dietary supplements that promote healthy aging are mostly warranted in an aging society. Because of age-related risks, anti-inflammatory and anti-oxidative agents such as microalgae are potential candidates for intervention. In a randomized controlled trial, we tested *Phaeodactylum tricornutum* (PT), a microalgae rich in eicosapentaenoic acid (EPA), carotenoids, vitamins, and β-glucans, cultured in bioreactors. In this pilot trial, 19 healthy elderly received supplements for two weeks based on either the whole PT (*A*), the β-1,3-glucan-rich PT supernatant (*SupB*), the combination thereof (*A+SupB*), or a Comparator product (*Comp*). The primary outcome variable plasma interleukin-6 was reduced after treatment with *A+SupB* compared to the *Comp* group (*p* = 0.04). The mobility parameters 5 s sit-to-stand test (*p* = 0.04 in the *A* group) and by trend gait speed (*p* = 0.08 in the *A+SupB* diet) were improved compared to *Comp*. No treatment effects were observed for fatty acids, compared to *Comp* but omega-6 to -3 fatty acid ratio (*p* = 0.006) and arachidonic acid/EPA ratio (*p* = 0.006) were reduced within group *A+SupB*. Further, the *SupB* study product reduced faecal zonulin (*p* = 0.03) compared to the *Comp*. The data revealed an anti-inflammatory and potentially anti-oxidative effect of particular PT preparations, suggesting that they might be suitable for effects in healthy elderly.

## 1. Introduction

The WHO declares the aging of the world population as the most important demographic problem worldwide. Therefore, healthy aging is becoming increasingly important to maintain functional abilities [1]. Aging is accompanied by immune system dysfunction, changes in intestinal epithelial barriers [2,3] chronic inflammation, and an increase in oxidative stress due to the imbalance between pro- and antioxidant species [4]. Due to these age-related changes, often named “inflammageing”, microalgae have been proposed as an aid in the prevention of inflammation as their functional constituents exert multiple pharmaceutical and nutraceutical bioactivities. Microalgae contain long-chain omega-3 fatty acids (n−3 FA), especially EPA (20:5 n−3) and docosahexaenoic acid (DHA, 22:6 n−3), otherwise found in fish, but also proteins, phenols, carotenoids, vitamins and dietary fibres such as β-glucans, in particular chrysolaminarin [5,6,7]. The microalgae *Phaeodactylum tricornutum* (PT) is a microalgae of particular interest because it is a species highly effective in synthesizing EPA, proteins, fucoxanthin and chrysolaminarin, and therefore a candidate for promoting healthy aging with preventive mechanisms. Compared to sea fish, the consumption of PT could be a sustainable nutritional alternative, as shown in previous studies from our and other groups [8,9]. By replacing sea fish consumption with microalgae such as PT the problem of overfishing of the oceans worldwide could be reduced [10]. Also, for vegans, PT could be of interest, because it offers a high content of EPA, which is available in plant food only through α-linolenic acid (ALA) known to be poorly converted into EPA in the human organism [11]. EPA plays a central role in the production of anti-inflammatory eicosanoids, cytokines and the reduction of reactive oxygen species [12]. This anti-inflammatory effect has already been demonstrated for PT [13] and could help tackle inflammageing in the elderly. Furthermore, adequate protein supply in old age is crucial due to muscle loss and the risk of sarcopenia [14]. PT contains up to 60% of protein and therefore can also serve as a valuable protein source for the elderly population. A preclinical study showed that 48% of protein could be replaced by PT without adverse effects on bioavailability [15]. Further functional compounds are phenols and carotenoids, which act as antioxidants by scavenging free radicals [16], and also the neuroprotective effects of carotenoids might be beneficial for healthy aging [17]. The most abundant carotenoid in PT is the xanthophyll fucoxanthin (Fx), a carotenoid without provitamin A activity, that is found in brown-coloured microalgae and macroalgae such as seaweeds [18,19]. Fx is converted into fucoxanthinol (FxOH) and amarouxiaxanthin A (A × A), which has already been demonstrated for PT in a clinical study [8]. Consumption of Fx may have health benefits for older people, such as neuroprotective effects [20], antioxidant and antiproliferative effects [21]. PT contains other carotenoids such as lycopene, which have antioxidant effects [22]. Also, the β-carotene with its pro-vitamin A activity is important for the visual process [23], and has antioxidant potential [24], and could provide further health benefits for the elderly. A further relevant nutrient is tocopherol, amounts of which depend on the growing conditions, and is a powerful antioxidant which could have a protective role in aging [25]. Another component of PT is the β-glucan (β-G) chrysolaminarin (Chrl), a water-soluble β-(1,3)/-(1,6)-glucan (11:1), which is produced in higher concentrations under nitrogen-limiting conditions [26]. The safe and potential gut health effect was shown in a pre-clinical study by the intake of the whole β-glucan- rich PT as well the EPA-rich biomass [27]. Further immunomodulatory properties and increased antioxidant status could already be shown in fish [28,29]. This regulation of antioxidant status and intestinal barrier function could benefit health, especially in older people.

Considering this background, we performed a randomized, controlled pilot trial in older individuals who received different preparations of the microalgae PT or a comparator product (*Comp*), which is considered a Placebo consisting of only vegetarian bouillon powder for two weeks to assess safety aspects, the bioavailability of selected ingredients and effects on biomarkers related to healthy aging.

## 2. Results

### 2.1. Analysis of the Phenolic Content and Oxidative Potential of the Intervention Products

**Figure 1:**
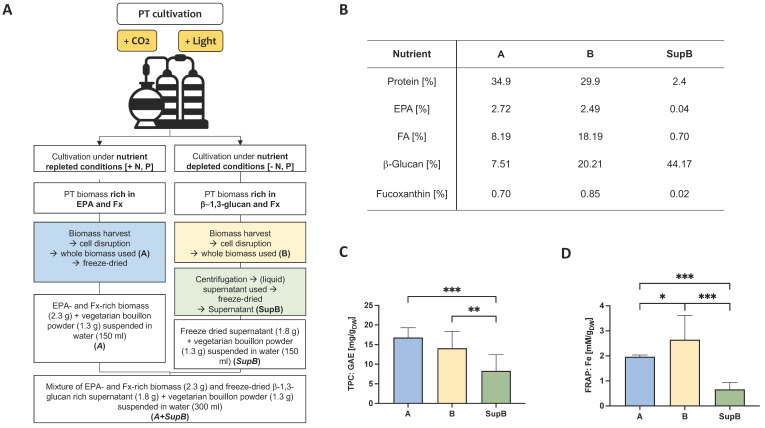
Different growing conditions were used for *Phaeodactylum tricornutum* (PT) production. Biomass A was grown under nutrient-repleted conditions leading to an EPA- and FX-rich biomass, whereas biomass B was grown under nutrient-depleted conditions (without nitrogen and phosphorous in the culture media) for several days before harvesting, leading to the accumulation of β-1,3-glucan. From biomass B, a supernatant was prepared (SupB). Based on biomass A and supernatant SupB, three supplements were prepared for the human trial (**panel A**). The nutrient composition of biomass A, biomass B and supernatant SupB differed (**panel B**). Total phenolics content (TPC) expressed as gallic acid equivalents (GAE) and ferric reducing antioxidant power (FRAP) were measured (**panels C**,**D**). Data are presented as mean ± SD (*n* = 3). Statistics: * indicates significant differences (ANOVA with Tukey post hoc test). * *p* < 0.05, ** *p* < 0.01, *** *p* < 0.001. Further abbreviations: N, nitrogen; P, phosphor; EPA, eicosapentaenoic acid; Fx, fucoxanthin; FA, fatty acids; DW, dry weight.

The production of PT with different cultivation conditions resulted in changes in the composition of ingredients (Figure 1A,B). Under nutrient-repleted conditions, PT accumulated more EPA (Biomass A). Under nutrient-depleted conditions, Chrl-rich biomass (Biomass B) was produced. From biomass B, a supernatant was prepared (for details see 4.3) and analysed as well as biomass A and B for the total phenolic content (TPC) by measuring the gallic acid equivalents (GAE; Figure 1C). The TPC ranged from 8.31 ± 4.13 mg GAE per g dry weight in the SupB, to 14.07 ± 4.34 mg/g within biomass B and 16.82 ± 2.47 mg/g in biomass A. The whole biomass samples A and B had a higher TPC compared to SupB (*p* < 0.001 for A, *p* = 0.009 for B). To analyse the antioxidant potential, the ferric reducing ability of plasma (FRAP) assay was performed (Figure 1D). The highest FRAP value was measured for biomass B (2.65 ± 0.97 mmol FRAP per g dry weight), which was higher than for biomass A (1.97 ± 0.07 mmol/g; *p* = 0.0480) and the SupB (0.66 ± 0.27 mmol/g; *p* < 0.001). The FRAP value was also higher in the A biomass compared to the Sup (*p* = 0.002).

### 2.2. Clinical Trial-Subjects’ Anthropometric and Metabolic Characteristics at Baseline

**Figure 2:**
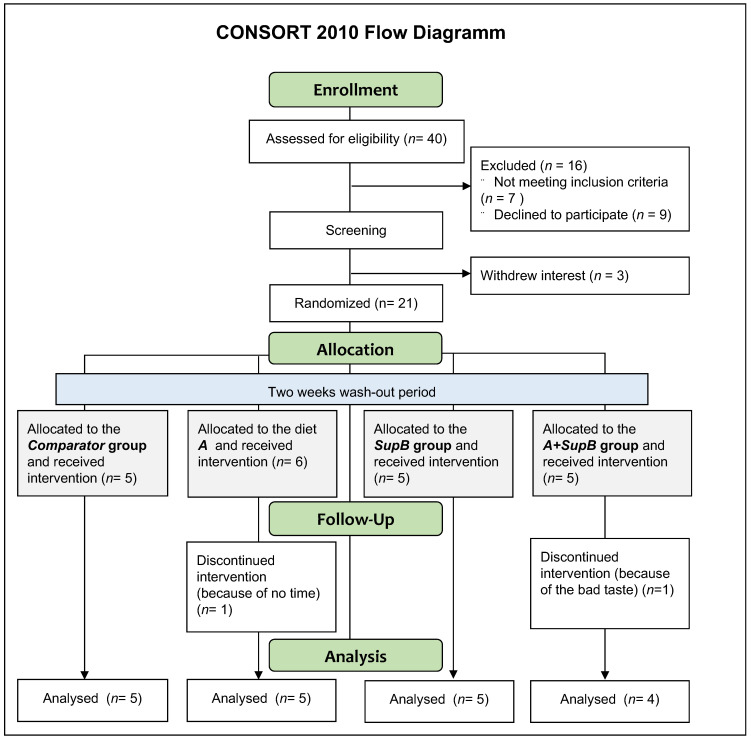
The study was designed as a pilot, randomized, single-blind, placebo (*Comp*)-controlled, parallel four-arm group study with a two-week wash-out and two-week intervention phase. After enrolment and screening (study start/time-point (0), a total of 21 participants were randomly assigned to four groups and underwent a two-week wash-out phase with dietary instructions. After these two weeks (time-point 2), they returned and received the study products assigned to their group. After one week of intervention (time-point 3), the third visit took place and two participants dropped out of the study. After two weeks of intervention (time-point 4), the fourth visit was conducted. The study was fully completed by 19 participants, which were analysed. Abbreviations: *Comparator*, is considered a Placebo with only vegetarian bouillon powder; *A*, diet with biomass A and vegetarian bouillon powder; *SupB*, diet with supernatant (*SupB*) of biomass B and vegetarian bouillon powder; *A+SupB*, Intervention with biomass A and supernatant of biomass B and vegetarian bouillon powder. For details of study products labelled with italic abbreviations (to separate it from biomass A and B in Figure 1) see chapter 4.3 and Table S7.

Recruitment of study participants took place between June 2021 and August 2021. After the study entry and during the study, there were a total of three dropouts for personal reasons after screening (Figure 2). Thus 21 subjects were randomized into four study groups. Two additional subjects dropped out during the intervention phase due to lack of compliance, so 19 individuals could be finally analysed (12 females, 7 males). The mean (±SD) overall age of the subjects was 67.7 ± 6.5 years, with nine (37.5%) being ≥70 years old (Table 1). The participants were at the normal weight on average with a mean BMI of 24.5 ± 3.1 kg/m^2^. Laboratory parameters did not show abnormal values except higher serum fat values than the reference values (cholesterol <200 mg/dl, LDL < 130 mg/dl, HOMA-Index < 1). There was no difference between study groups except the waist circumference was different within the groups (*p* < 0.001).

The study population was divided into four groups that received different supplements, (i) a Comparator (*Comp*) consisting of a vegetarian bouillon powder solved in 150 mL plain water (*Comp*), (ii) lyophilizate of PT biomass A mixed with bouillon powder dissolved in water (*A*), (iii) lyophilizate of supernatant of PT biomass B mixed with bouillon powder solved in water (*SupB*), (iv) a combination of (ii) and (iii) (*A+SupB*). For details of study products labelled with italic abbreviations (to separate it from biomass A and B in Figure 1) see chapter 4.3 and the nutrient composition in Table S7. 

### 2.3. Four-Week Food Diary and Food Frequency Questionnaire (FFQ)

Diet was assessed using an FFQ at the study start and a continuous food diary during the intervention period as described in Methods 4.10. The FFQ shows similar dietary intake between study groups (Table S1). The food diaries allowed for analysing the percentage compliance with recommended nutrient amounts for the corresponding age group, either 51–65 years or > 65 years. On average, the recommendations were exceeded for protein (124%), PUFA (151%), and vitamin A (257%) and were not fully met for energy (90%), carbohydrate (65%), fibre (73%) and vitamin D (9%) intakes.

### 2.4. Acceptance and Adverse Effects during the Intervention

All four study products were generally well tolerated. Adverse effects were monitored using a diary and at each study visit by the study staff (protocol). No serious adverse reactions were reported, but minimal and mild adverse effects occurred (Table 2). Most side effects occurred after taking *A+SupB*, e.g., belching, headache and increased urination. Gastrointestinal symptoms such as abdominal rumbling, flatulence and diarrhoea were described for all four treatments as well as for the *Comp*.

### 2.5. Effect of Supplementation on Laboratory Parameters

**Figure 3:**
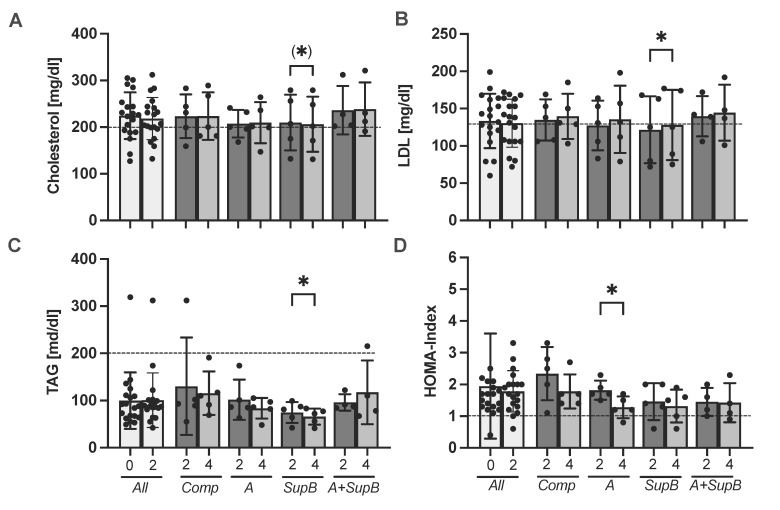
Effect of the study products on total serum cholesterol (**A**), LDL-cholesterol (**B**), triacylglycerols (**C**) and HOMA index (**D**). Parameters were measured at study start (0), after two-weeks wash-out (2), and after two weeks of intervention (4). Values are expressed in scatter plot with mean ± SD and individual values from 19 participants (*All* *n* = 19; *Comp*, *A*, *SupB* each *n* = 5; *A+SupB n*= 4). Below the dashed line is the normal reference range. Statistics: * indicates a difference within a group between week two (2) and week four (4) (t-test). No between-groups-differences were found at week 0, 2 and 4 (ANOVA). (*) *p* < 0.1, * *p* < 0.05. Abbreviations: diets see Figure 2; *Comp*, Comparator; LDL, Low-density lipoprotein; TAG, Triacylglycerols; HOMA index, Homeostasis Model Assessment for Insulin Resistance.

Various laboratory parameters were determined at three time points at study start (0), after the wash-out phase (2) and after two weeks of intervention (4). Half of the subjects cholesterol levels were over the normal ratio. Within the *SupB* group, the cholesterol levels were lower by a trend at week four (206.2 ± 59.1 mg/dl) compared to before the intervention (209.8 ± 59.6 mg/dl; *p*= 0.06; Figure 3A). The LDL was higher within the *SupB* group at week 4 compared to week 2 (*p*= 0.04, Figure 3B). Triacylglycerols (TAG) were reduced at week 4 with 65.8 ± 17.1 mg/dl compared to week 2 (74.6 ± 22.2 mg/dl; *p* = 0.04, Figure 3C). Within the A group, the Homeostasis Model Assessment (HOMA) index for insulin resistance was higher at week two at 1.82 ± 0.3 compared to 1.28 ± 0.3 at week 4 (*p*= 0.03, Figure 3D). Further laboratory parameters are shown in Table S2. No difference was measured between groups and the change from week 4 to 2.

### 2.6. Mobility Markers and Body Composition before and after Intervention

**Figure 4:**
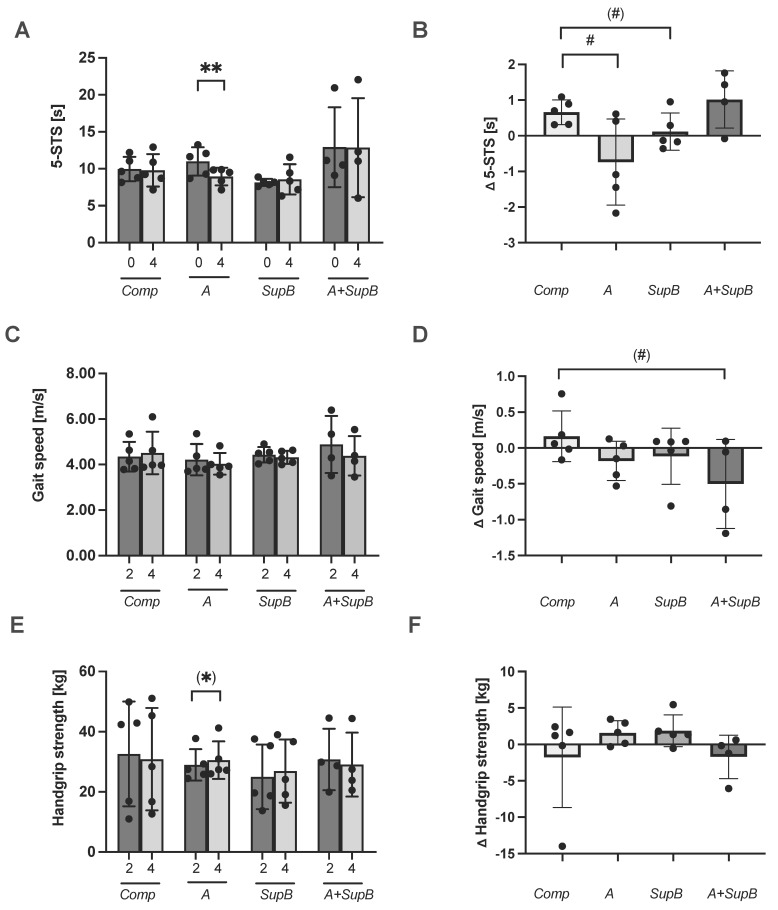
Effect of study products on the mobility markers gait speed (**panels A**,**B**), the 5 s sit-to-stand test (5-STS; **panels C**,**D**) and handgrip strength (**panels E**,**F**). Data at study start (0) and after (4) intervention (panel A), week 2 and 4 (**panels C**,**E**) and treatment effect expressed as differences between weeks 4 and 2 (Δ) (**panels B**,**D**,**F**) are shown. Values are expressed in scatter plots with mean *± SD* and individual values from 19 participants (*All* *n* = 19; *Comp*, *A*, *SupB* each *n* = 5; *A+SupB n*= 4). Statistics: * indicate a difference within a group between study start (0) and week four (4) (t-test); ^#^ indicate a difference between groups (ANOVA). (*/^#^) *p* < 0.1, ^#^ *p* < 0.05, ** *p* < 0.01. Abbreviations: diets see Figure 2 *Comp*, Comparator.

The Western Ontario and McMaster Universities Arthritis Index (WOMAC) questionnaire was used to test the mobility of the knee and hip, including pain, stiffness, and physical functioning. There was no treatment effect measured (Table S7). The index was 9.2 ± 10.4 points at the study start and 11.1 ± 13.9 at study end. Within groups it varied at study end between 2.8 ± 3.0 points in the *A* group and 16.3± 18.9 in the *A+SupB* group. The time for the 5 s sit-to-stand test (5-STS) showed a reduction in the time from 11 ± 1.9 sec to 9.0 ± 1.2 sec within the *A* group from 0 to week 4 (*p* = 0.02; Figure 4A). The delta was lower by −0.7 sec within the *A* group which was significantly lower compared to the *Comp* group (*p* = 0.04; Figure 4B). Measurement of the gait speed test showed no changes within groups (Figure 4C), but there was a trend of a treatment effect by the delta reduction within the *A+SupB* group compared to the *Comp* group (*p* = 0.08, Figure 4D). The handgrip strength tended to increase in the A group from 29.0 ± 5.2 kg at week 2 to 30.6 ± 6.3 kg at week 4 (*p* = 0.09, Figure 4E) but there was no delta change (Figure 4F). Body composition markers were not affected by the study interventions (Table S3) except the lean body mass was lowered after the *SupB* study product and by a trend of the others compared to the *Comp*. 

### 2.7. Fatty acid Changes in Plasma and Erythrocyte Membrane/Red Blood Cells (RBC)

**Figure 5:**
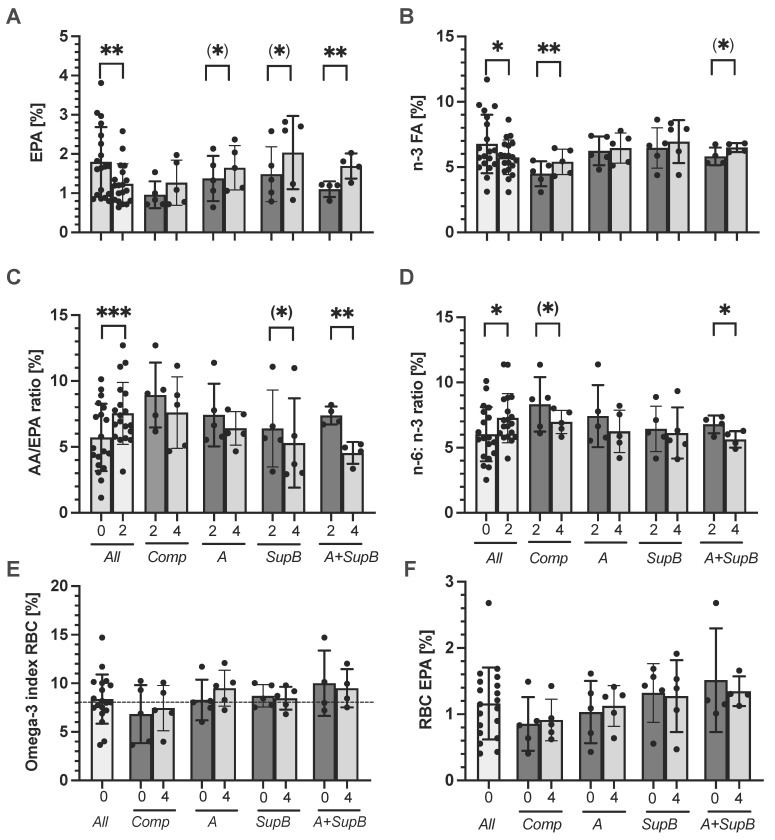
Effect of study products on the plasma fatty acid (FA) levels eicosapentaenoic acid (EPA; **panel A**), omega-3 fatty acids (n−3 FA; **panel B**), arachidonic acid (AA)/EPA ratio (**panel C**) and n−6 FA to n−3 FA ratio (**panel D**). Parameters were measured at study start (0), week two (2) and week four after the intervention (4). Further, red blood cell (RBC) FA were measured, namely the omega-3 index (**panel E**; marked threshold value at 8%) and the RBC EPA content (**panel F**). Values are expressed in scatter plots with mean, *SD* and individual values from 19 participants (*All n* = 19; *Comp*, *A*, *SupB* each *n* = 5; *A+SupB n* = 4). Statistics: * indicate a difference within a group between week two (2) and week four (4) (t-test). No between-groups-differences were found at week 0, 2 and 4 (ANOVA). (*) *p* < 0.1, * *p* < 0.05, ** *p* < 0.01., *** *p* < 0.001. Abbreviations: diets see Figure 2; *Comp*, Comparator.

**Figure 6:**
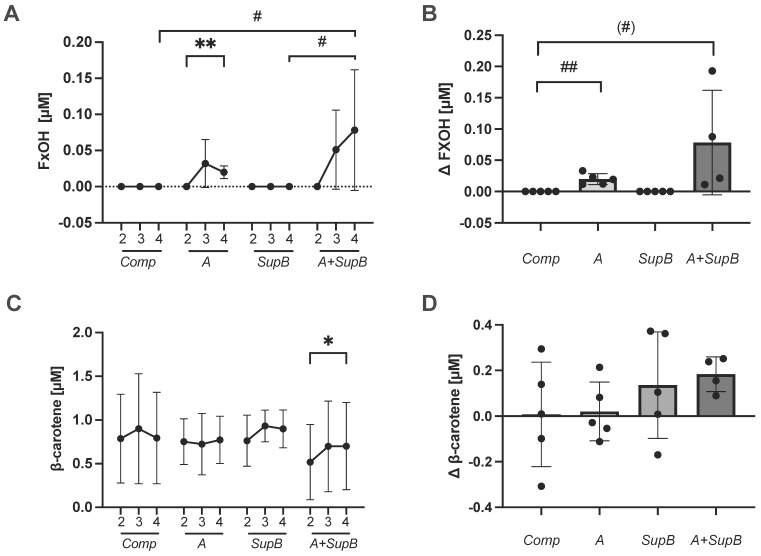
Plasma carotenoids changes following interventions. Fucoxanthinol (FxOH, **panels A**,**B**) and β-carotene (**panels C**,**D**) were shown before the intervention (2), after week three and one week of supplementation (3) and week 4 after two weeks of supplementation (4) (**panels A**,**C**). The treatment effect expressed as differences between weeks 4 and 2 (Δ; **panels B** and **D**) with *Comp, A, SupB,* and *A+SupB*. Values are expressed as mean and SD error bars. Statistics: *indicate a difference within a group between week two (2) and week four (4) (t-test); ^#^ indicate a difference between groups (ANOVA). (^#^) *p* < 0.1, */^#^ *p* < 0.05, **/^##^ *p* < 0.01. Abbreviations: diets see Figure 2; *Comp*, Ccomparator.

The plasma FA levels were determined at study start (0), after the wash-out phase (2) and after two weeks of intervention (4) (Figure 5 and Table S4). Comparing all plasma data at the study start (0) with after the washout period (2), when no fish was consumed, plasma levels of EPA (*p* = 0.005; Figure 5A), n−3 FA (*p* = 0.02; Figure 5B), DHA (*p* = 0.008), EPA + DHA (*p* = 0.005) decreased and others by a trend (Table S4). Further, the AA/EPA ratio increased (*p* < 0.001; Figure 5C) and n−6:n−3 ratio (*p* = 0.01; Figure 5D) after the washout period compared to the study start (Table S4). 

Regarding within-group differences from week 2 to week 4, the EPA plasma concentrations tended to increase by 0.3% within the *A* group and 0.55% within the *SupB* group (*p* = 0.1; Figure 5A). Only within the *A+SupB* group, the EPA level increased significantly by 0.6% (*p* = 0.006). The DHA level did not change due to the intervention (Table S4). The EPA + DHA level did not change except for an increase within the *Comp* group (Table S4). Total n−3 FA plasma concentration increased within all interventions but only significantly within the *Comp* group by 0.9% (*p* = 0.005) and by a trend within the *A+SupB* group by 0.7% (*p* = 0.05) (Figure 5B). The AA/EPA ratio decreased from week 2 to week 4 after all supplementations but only significantly within the *A+SupB* group from 7.4 ± 0.7% to 4.5 ± 0.8% (*p* = 0.006; Figure 5C) and by a trend within the *SupB* group (*p* = 0.09). Furthermore, the n−6:n−3 ratio was lower within the *A+SupB* group at week four with 5.6 ± 0.6% compared to week two with 6.8 ± 0.1% (*p* = 0.006, Figure 5D). A reducing trend was further measured within the *Comp* group (*p* < 0.01). Minor other changes in plasma FAs were measured as shown in the Supplementary Materials in Table S4. Regarding treatment changes, which were calculated as the difference between week 4 to 2, the 18:3n−3 FA level was lowered after the *A* diet compared to the *Comp* group (*p* = 0.008). The 22:6n−3 level in the *SupB* group (*p* = 0.02) and the 20:3n−6 level within the *A* group were reduced compared to the *Comp* group (*p*= 0.05; Table S4).

The RBC FA concentration was measured at the study start (0) and after 4 weeks. The mean total Omega-3 index at the beginning was 8.4 ± 2.5% (Figure 5E), consisting of levels of EPA (1.16 ± 0.5%, Figure 5F) and DHA (7.21 ± 2.1%). Within the *A* group, the index changed from the study start from 8.29 ± 2.1% to 9.49 ± 1.9%, but not significantly, and no changes were measured comparing both time points. 

### 2.8. Carotenoids, Vitamin E Changes in Plasma 

Due to the carotenoid and tocopherol concentrations in PT, the amounts in the blood plasma were measured at four different time points and shown in Figure 6A and C at weeks 2, 3 and 4 and the delta between weeks 2 and 4. The *A* and *A+SupB* diets contained Fx in the diet and the participants took 21.4 mg or 21.6 mg daily. After one week of intake at week 3, no Fx could be measured in the blood plasma of the subjects. FxOH was detected after one week (3) (0.03 ± 0.03 µM) and two weeks (4) of *A* diet intake (0.02 ± 0.001 µM) and the level significantly increased from week 2 to 4(*p* = 0.007, Figure 6A). Within *A+SupB* group, FxOH was detected after one week (3) (0.05 ± 0.05 µM) and two weeks (4) (0.08 ± 0.08) in the plasma. The change between weeks 4 to 2 (Δ) for FxOH, was higher in group *A* (*p* = 0.001; Figure 6B) and *A+SupB* group by a trend compared to the *Comp* group. A × A, to which FxOH is converted in the liver [30], could be measured in plasma at 0.003 ± 0.006 µM in only one participant after the study product *A+SupB*. Through the *A* and *A+SupB* diets, subjects consumed 0.3g of β- carotene daily. After two weeks of supplementation, plasma β-carotene increased from 0.5 to 0.7 µM. (*p* = 0.02, Figure 6C) within the *A+SupB* group. No changes between week 4 to 2 were measured (Figure 6D, Table S5). Further, carotenoids such as retinol did not change in any group, nor did the lycopene plasma levels. Furthermore, no increase in γ-tocopherol and α-tocopherol were measured (Table S5). 

### 2.9. Inflammatory Parameters and the XOR as an Oxidative Stress Marker

**Figure 7:**
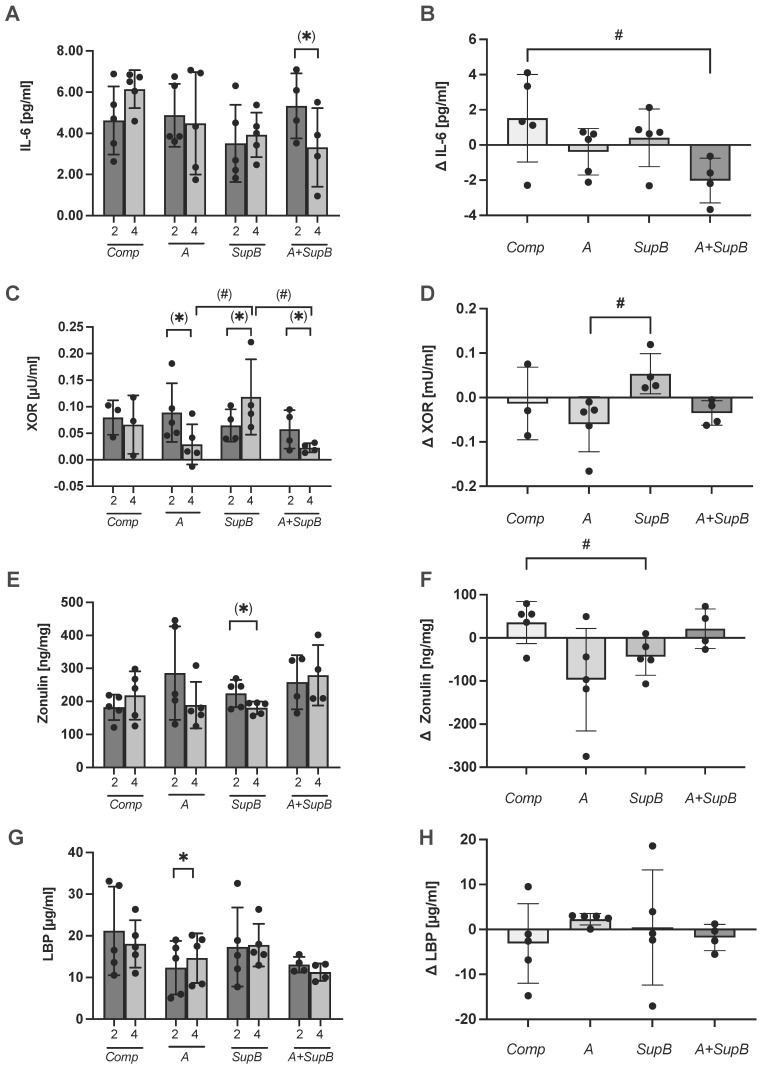
Effect of the study products on plasma interleukin-(IL)-6 (**panels A**,**B**), plasma xanthin oxidoreductase (XOR; **panels C**,**D**), and the gut barrier markers faecal zonulin (**panels E**,**F**) and plasma lipopolysaccharide-binding protein (LBP) in plasma (**panels G**,**H**). Data before (2) and after (4) intervention (**panels A**,**C**,**E**,**G**) and treatment effect expressed as differences between weeks 4 and 2 (Δ) (**panels B**,**D**,**F**,**H**) are shown. Values are expressed in a scatter plot with mean and *SD*-individual values from 19 participants (*Comp* , *A*, *SupB* each *n* = 5; *A+SupB n* = 4). Statistics: *indicates a difference within a group between week two (2) and week four (4) (t-test); ^#^ indicates a difference between groups (ANOVA). (*/^#^) *p* < 0.1, */^#^ *p* < 0.05. Abbreviations: diets see Figure 2; *Comp*, Comparator.

A two-week intervention with *A* and *SupB* had no negative effect on inflammatory markers such as high-sensitivity C-reactive protein (hs-CRP), interleukin (IL)-10, IL-6 and tumour necrosis factor (TNF)-α. Comparing before (2) and after the intervention (4) within the group of *A+SupB* a trend was shown to decrease the pro-inflammatory marker IL-6 level from 5.3 ± 1.6 pg/mL at week two to 3.3 ± 1.9 pg/mL at week four (*p* = 0.5; Figure 7A). The treatment effect measured as the change of weeks four to two, showed a significant difference between the *A+SupB* group (−2.0 ± 1.3 pg/mL) and the *Comp* group (+1.5 ± 2.5 pg/mL; *p* = 0.042; Figure 7B). The hs-CRP, IL-10 and TNF-α did not change according to the diet or differ between the diets. As an oxidative stress parameter, the xanthine oxidoreductase (XOR) was measured. The XOR is the final enzyme in purine catabolism and catalyses hydroxylation to xanthine and then to uric acid. The results showed a tendency to decrease levels within the *A* and *A+SupB* groups compared to the levels before the intervention (2) and after intervention (4) (*p* < 0.1). The XOR tended to increase within the *SupB* group (Figure 7C). The treatment effect showed no effect compared to the *Comp* group only between *A* and *SupB* (Figure 7D). 

### 2.10. Gut Barrier Markers and Short-Chain Fatty Acids

The gut barrier marker lipopolysaccharide-binding protein (LBP), an acute-phase protein that binds to bacterial lipopolysaccharides derived in part from translocation from the intestine and zonulin, which is an acute-phase response protein and controls intestinal permeability by reducing the stability of tight junctions (TJ), revealed small changes within the different groups. The faecal zonulin showed a trend of reduction within the *SupB* group comparing the level of 223.8 ± 41.5 ng/mg at week 2 to 180.3 ± 19.3 ng/mg at week 4 (*p* = 0.088; Figure 7E) and a treatment effect compared to the *Comp* group (*p* = 0.03; Figure 7F). The LBP was higher at week 4 with 14.6 ± 5.9 µg/mL within the *A* group compared to 12.4 ± 6.4 µg/mL at week 2 (*p* = 0.045; Figure 7G) but no treatment effect was measured compared to the *Comp* group (Figure 7H). SCFA concentrations in faeces showed no significant changes between groups or due to the interventions (Table S6).

### 2.11. Correlations

**Figure 8:**
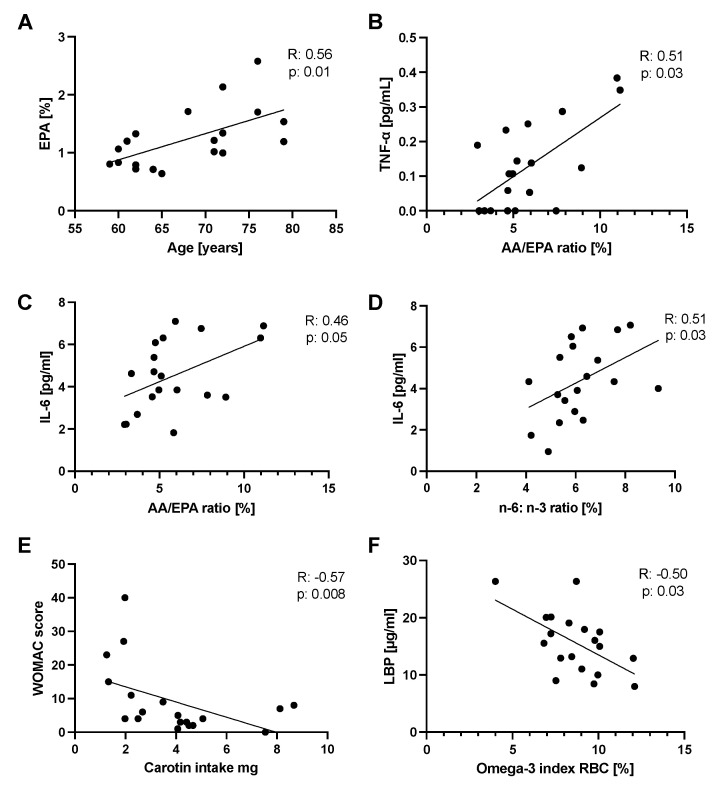
Positive correlations were shown by Spearman (R) correlation as the EPA plasma concentration (week 2) to the age (**panel A**), AA/EPA plasma ratio to the TNF-α (week 4) (**panel B**), Il-6 to the AA/EPA ratio (week 4) (**panel C**), and the n−6: n−3 ratio (week 4) (**panel D**). A negative association was found between the WOMAC score and the carotenoid intake measured by the FFQ (**panel E**) and the LBP level and the omega-3 index (week 4, **panel F**). Statistics: R, Spearman rho; *p*-values > 0.05. Abbreviations: EPA, eicosapentaenoic acid; AA/EPA, arachidonic- and eicosapentaenoic acid ratio; n−6: n−3, n−6 FA to n−3 FA ratio; TNF-a; tumour necrosis factor; IL-6, interleukin-6; WOMAC, Western Ontario and McMaster Osteoarthritis Index; LBP, lipopolysaccharide-binding protein; RBC, red blood cells (erythrocyte).

Spearman correlation showed a positive association between different FAs, especially EPA and the age of the participants (Figure 8A). A positive correlation was measured between the AA/EPA plasma ratio with TNF-α and IL-6 (Figure 8B,C) and the n−6: n−3 ratio with IL-6 (Figure 8D). A negative association was found between the WOMAC score and the carotenoid intake measured by the FFQ (Figure 8E). Regarding the gut barrier marker LBP, a negative association with the omega-3 Index was measured (Figure 8F). 

## 3. Discussion

The present study investigated the anti-aging effect of different preparations of the microalgae PT and Comparator in a randomized controlled manner. So far, PT has not been approved as a novel food; therefore, the safe intake, as well as possible health promotion effects, were investigated.

### 3.1. Safety and Bioavailability

Microalgae are not yet widespread on the food market and are mostly sold in capsules, tablets, or dried powder as dietary supplements. A new approach is to use the whole microalgae and convert it into novel and tasty food products with potential health-beneficial effects. The incorporation of microalgae biomass into foods has encountered some difficulties mainly due to the colour and fishy taste [31]. PT with its unique content of functional nutrients is particularly difficult to process due to its intense brown/green colouration and the oxidation of FA and carotenoids, which favours the fishy taste [32]. However, due to the combination with the vegetarian bouillon powder, the ingestion became acceptable by most study participants, although other studies rated the taste fishy and unpleasant [33]. The general intake of the β-G-rich supernatant (*SupB*) was very well accepted as it was almost tasteless when dissolved in water. Previously, it was shown that β-Gs contained in beverages and liquid test meals turned out to be the best carriers [34], as it was used in the present study.

Our study provides further evidence for safe intake by evaluating several laboratory parameters. Except for the *SupB* group, which showed an increase in LDL-cholesterol, but still within the normal reference range, no negative effects on laboratory parameters were observed. As we could not measure treatment effects compared to the *Comp*, the results still suggest health-promoting effects within the groups, such as the reduction in HOMA-index within the *A* group after the whole PT supplementation. n−3 FA could generally improve insulin sensitivity, although these results are mostly based on animal models [35]. Further n−3 FAs have been reported to lower TAG, which may lead to a reduction of the risk of cardiovascular diseases (CVD) [36]. In our study, such results could not confirm after PT supplementations (*A* or *A+SupB* diets), possibly because it was underpowered for such an effect. However, a pre-clinical study in Wistar rats fed a high-fat (HF) diet supplemented with 12% PT already shows a TAG and HOMA-index lowering effect compared to the HF diet without PT supplementation [37]. 

A reduction of cholesterol by a trend and TAG levels was measured within the *SupB* group, after supplementation with the Chrl-rich supernatant. Although our data could not confirm previous pre-clinical studies with zebrafish showing cholesterol-lowering and LDL-lowering effects following PT supernatant supplementation similar to *SupB* [38], they did suggest a possible trend. Especially in older people, in whom lipid metabolism is frequently altered [39], LDL cholesterol-lowering could be a valuable goal, since LDL cholesterol is a modifiable cardiovascular risk factor for prevention [40]. Meta-analyses have already demonstrated the β-G cholesterol-reducing and LDL-lowering effect of taking a dose >3 g of β-G per day by consuming oats and barley for at least three weeks in individuals with mild hypercholesterolemia [34]. In our study, we administered only about 0.5 g of β-G per day, which might be suboptimal. A higher supplementation of the Chrl-rich supernatant (*SupB*) could produce greater effects, which needs to be confirmed in future trials. In a pre-clinical study, the safe intake of up to 4621 mg/kg body weight of the β-G Chrl in mice has been shown [27]. For human nutrition, β-Gs are an important dietary fibre supporting preventive effects [41], and microalgae containing β-Gs like *Odontalla auritia* and *Euglena gracilis*, have already been approved by the European Food Safety Authority (EFSA). Since the recommended daily fibre levels were not reached by the participants, additional supplementation with PT Chrl-rich supernatant would be conceivable for older people and others.

In terms of FA bioavailability, the wash-out phase was effective due to the plasma FA reduction. Further, an increase of EPA was expected after the PT diets *A* and *A+SupB,* comparable to the previous study with younger participants [8]. However, no treatment effect was observed. Our study could not confirm such findings, possibly because it was underpowered. Age differences might be related to altered FA metabolism in older people, such as slower plasma clearance and lower incorporation into cell membranes [42], as well as changes related to FA release and/or β-oxidation in older people [39]. However, our present data showed that plasma n−3 FA levels were already higher in the older participants before the study than in the younger study participants [8]. This is consistent with other studies [43,44] and with our correlation analyses, which showed higher plasma EPA levels with increasing age. In addition, nutritional data also show a higher EPA+DHA intake in older women and men (65–79 years) at 232.1/277 mg/day (women/men) compared to 18–24-year-olds at 199.9/232.1 mg/day (women/men) [45]. The general recommended intake of EPA+DHA is 250–500 mg daily and might be even higher for some prevention goals [46], suggesting that no age group is meeting the recommendations. Therefore, PT could be an additional dietary source of EPA-FA. Measurement of the Omega-3 index in erythrocytes was performed to verify adequate supply. This varied between 6.8% and 10%, confirming a good supply and moderate to low risk for CVD in our study population [47]. As the Omega-3 index measured in RBC is considered a marker for long-term FA changes, no changes were expected and observed in our study after two weeks of PT supplementations. Our present study results indeed suggest a possible altered FA metabolism in older adults. Therefore, the recommended intake of EPA and DHA should be reconsidered and possibly adjusted in future for the elderly population.

Regarding the uptake of carotenoids, the present study confirms the metabolization of Fx to FxOH, as previously shown [8]. Further metabolization to A × A could only be measured in one subject. This seems to be dependent on the ingested amount of Fx, since a previously conducted study with supplementation of about 30mg Fx per day led to a significant increase of both metabolites [8]. So far, the EFSA recommends an amount of 15 mg of pure Fx per day, e.g., derived from *Undaria pinnatifida thallus* extract (wakame) [48]. Despite several possible health benefits, no health claims have been awarded so far for Fx-containing microalgae [49,50]. Future studies not only on health benefits but also regarding dose-finding and toxicity levels are needed to promote the acceptance of Fx as a valuable food supplement in future.

For tocopherol and β-carotene, PT might not be the best selection, because their content is rather low and therefore would require an intake of higher amounts of the microalgae than those chosen in our study. This might explain why we found no plasma increase of tocopherol and only some increase of β-carotene in the *A+SupB* group. This group tended to have the lowest baseline levels, suggesting that PT supplementation can increase plasma β-carotene levels as recently demonstrated [8]. 

In terms of gut health, minimal and mild side effects were noted after ingestion of the study products, as reported earlier [8]. Because loss of gut barrier function [2] and changes in the gut microbiome [51,52] may occur with aging, we were interested in whether PT supplementation could prevent such alterations in the study population. The gut barrier markers plasma LBP and faecal zonulin were measured to assess intestinal barrier function. The results yielded a treatment effect of zonulin, which decreases within the *SupB* group compared to the *Comp* group. Since PT contains high amounts of n−3 PUFAs, which are thought to promote the intestinal barrier [53], we expected a treatment effect as well within the *A* and *A+SupB* groups. We found no such effect, but a negative correlation between the Omega-3 index in erythrocytes and plasma LBP levels. The reduction of faecal zonulin levels within the *SupB* group could also have implications beyond the gut since zonulin is positively correlated with the concentration of pro-inflammatory cytokines (TNF-α and IL-6) and negatively correlated with muscle strength and usual physical activity [54]. In a pre-clinical study supplementing the whole EPA-rich PT biomass and β-G-rich PT biomass, we found an increase in SCFA and SCFA-producing bacteria, suggesting possible healthy gut promoting effects [27]. However, in the present human trial, we could not confirm such findings, possibly because of smaller amounts of PT biomass administered based on a bodyweight-related dosage.

### 3.2. Potential Antioxidative and Anti-Inflammatory Effects

In terms of antioxidant potential in the PT biomass, the highest phenolic content was measured for both whole biomasses rich in EPA (A) and those rich in β-G Chrl (B) and half of the potential in the supernatant (Sup). The results show that for a higher phenolic content, the use of whole biomass is necessary. Previously, it was shown that PT contains fourteen phenolic compounds [55], which could be useful for human nutrition. Regarding the antioxidant potential measured by the FRAP assay, the highest value was measured for the β-G-rich PT biomass B. This biomass contained the highest Fx amount, which confirms that carotenoids and xanthophylls could have a high antioxidant potential [56]. The antioxidant effect of Fx extracts from PT has been reported previously [21]. However, the EPA-rich biomass A also exhibited antioxidant potential, as FAs may also have antioxidant activity [57]. 

Our human study provides the first evidence of a potential anti-oxidative effect of the EPA-rich PT biomass, as plasma XOR levels tend to be lower in group *A* and group *A+SupB*, which ingest the whole PT. In the *SupB* group, which ingested the Chrl-rich supernatant, this trend toward lowering was not observed. XOR levels are often elevated in inflammatory bowel diseases [58] and age is positively correlated with xanthine oxidase activity [59]. Therefore, the trend of reducing the XOR levels is a possible positive indication. Other studies with PT have already shown that the microalgae reduce the activity of nuclear factor kappa B (NF-κB) in mouse macrophages [13], which is activated by the production of reactive oxygen species (ROS). Moreover, it has been shown that the free radical scavenging activity is presumably related to the high Fx content of PT [30,60]. These findings are consistent with our results, as diets *A* and *A+SupB*, which have the highest Fx content, showed a trend for some XOR reduction within the groups. Not only Fx, but also n−3 FAs might have a stimulatory effect on mitochondrial function and fusion processes by reducing ROS production [61]. Higher doses and more participants are required to prove statistically significant effects.

To tackle inflammageing in elderly the current study shows potential anti-inflammatory treatment effects of the *A+SupB* group, by the reduction of the pro-inflammatory cytokine IL-6 compared to the *Comp* group. Other inflammatory parameters such as TNF-α and C-reactive protein remained unchanged. For PT, an anti-inflammatory effect has already been shown in vitro [13] and in vivo [37]. The effect could be addressed by the high Fx content, which regulates the NF-κB and NLRP3 inflammasome activation [50]. Other PT compounds such as polysaccharide and EPA could be further involved in the reduction of pro-inflammatory cytokines such as IL-6 [62,63]. A further anti-inflammatory indicator could be the reduced AA/EPA ratio and the n−6:n−3 ratio in plasma within the *A+SupB* group. Both are reliable indicators of nutritional status, and a higher n−6:n−3 ratio can lead to the development of various metabolic disorders [64]. Indeed, our correlation analyses confirm this hypothesis, as the AA/EPA ratio was positively associated with IL-6 and TNF-α levels and the n−6:n−3 ratio with IL-6 level.

Considering age-related muscle wasting, supplementation of PT could prevent such deficits and promote functional ability. If higher amounts of PT are administered, it could even be an additional source of protein. n−3 FAs are thought to have an anabolic effect [65] and indeed cause improvements in muscle strength and protein synthesis [65,66], however, other studies did not find such effects [67,68]. The current study showed some modest treatment effects after the PT supplementation in terms of improvement in mobility markers such as the 5-STSs in the *A* group and gait speed within the *A+SupB* group compared to the *Comp* group. Regarding the effects of carotenoids, higher intake is associated with better grip strength [69], and reduction of hip fractures [70], therefore PT in higher doses may be a valuable nutrient for older adults. Higher dietary intakes of α-tocopherol and lycopene are negatively associated with the WOMAC score (a higher score means more severe pain and functional limitations) [71]. The present data suggest this association, as a negative correlation was found between the WOMAC score and carotenoid intake. In addition, supplementation with n−3 FAs, carotenoids, and vitamin E has been found to improve working memory in older adults [72], which closely matches the constituent nutrients of PT and suggests a potential benefit in the elderly.

### 3.3. Limitations

The study shows limitations, as some effects such as cholesterol- and TAG-lowering effects are demonstrated only in the *SupB* group, but not in the *A+SupB* group, which consumed the same amount. It has been expected that the effects would occur in both groups. The findings from this pilot trial need confirmation from larger confirmatory trials. 

## 4. Materials and Methods

### 4.1. Participant Selection

Both females and males, aged between 60 to 90 years were screened with a BMI between 18.5 to 30 kg/m^2^ and a weight of more than 50 kg. Participants had to be willing to adhere to certain dietary rules (no significant changes in diet, no fish or seafood and no probiotics) and the willingness to follow the prescribed diet for the duration of the study (14 days). Physical activity should not be changed throughout the study. Exclusion criteria were the intake of intestinal therapeutics, antibiotics, immunosuppressants, cholesterol-lowering drugs or similar, relevant violations of the dietary protocol, occurrence of relevant diseases—diabetes mellitus, lipid metabolic disorders, severe acute COVID disease within the last six weeks according to a case-by-case decision, and acute COVID disease. Inclusion and exclusion criteria remained unchanged throughout the study. The study was conducted according to the Declaration of Helsinki at the Center for Clinical Nutrition Stuttgart at the University of Hohenheim in 2021 and has been approved by the local Ethical Committee (Ethik-Kommission der Landesärztekammer Baden-Württemberg, F-2021-061), and was registered at ClinicalTrials.gov (NCT05120791). 

### 4.2. Study Design and Outcome Parameters

The study was designed as a randomized, single-blind, 1:3 Comparator controlled (considered a Placebo with only vegetarian bouillon powder) parallel group with four visits (Figure 2). An intervention period of two weeks was chosen due to the increase in FA of the previous human study [8] and to estimate a safe intake of the β-G supernatant (*SupB*). When participants fulfilled all inclusion and no exclusion criteria at the study start, visit parameters were assessed. They were requested to eat no fish and seafood for all four weeks. The first two weeks were planned as a wash-out period for the n−3 FAs and the second visit was after two weeks (2). The third visit was after three weeks (3) and the fourth visit was after four weeks (4). The study was planned as a proof-of-principle pilot study; therefore, no formal calculation of case numbers was performed. 

The primary outcome parameter was the effect on inflammation markers (hs-CRP, IL-6, 10). Secondary outcome parameters were laboratory parameters, n−3 FA in plasma, erythrocyte/ RBC, the improvement of the n−6: n−3, and AA/EPA ratio, carotenoids, body weight, waist circumference, handgrip strength, BIA assessing, gut barrier marker (LBP, zonulin) and SCFAs. For muscle function 5-STS, gait speed, handgrip, and the WOMAC questionnaire were used. Diet was evaluated using a food diary and an FFQ. At the study entry, the subject got verbal and written informed consent and the inclusion and exclusion criteria were obtained. The demographic data were collected, the medical history and the collection of former and current medication. The bioelectrical impedance analysis and the 5-STS were done and the fasting venous blood collection for blood markers and FAs (plasma and RBC), as well as anthropometric measurements and hand strength were done at the institute. The nutrition diary, FFQ and the instructions for the faecal sample collection were handed out. 

At the second visit (after week 2) the products were distributed, and fasting blood samples were taken to analyse blood markers and FAs (plasma). Faecal samples were collected and used for SCFA measurement and barrier permeability. Further, the gait speed and the 5-STS were completed. Participants started to take the capsules after the second visit. In the third visit after three weeks (3), fasting blood samples were obtained for blood markers and FAs (plasma) and the instructions for the faecal sample collection were handed out. The fourth visit after four weeks (4) was the same as the study entry and on the second visit and participants returned their investigational products.

### 4.3. Study Products, Randomization and Blinding

The EPA and Fx-rich PT biomass (biomass A) was produced under nutrient-repleted conditions in flat panel airlift reactors. The biomass was harvested and concentrated via centrifugation to 250–270 g/L (Clara 20, Alfa Laval, Glinde, Germany) as described before [8]; see also Figure 1. The cells were disrupted using a bead mill (PML 20, Bühler, Uzwil, Switzerland), and freeze-dried (VaCo 5, Zirbus). For the generation of supernatant, PT biomass B was produced in flat panel airlift reactors under nutrient-depleted conditions (without nitrogen or phosphorous source in the culture media) for several days before harvesting. Harvesting and concentrating were performed by centrifugation to 250–270 g/L (Clara 20, Alfa Laval) as described before [8]. After cell disruption via bead milling (PML 20, Bühler), the biomass was centrifuged again, and the liquid supernatant was separated from the biomass pellet to obtain the supernatant (SupB). Afterwards, the supernatant was freeze-dried (Avanti J-26 XP, Beckman Coulter, Brea, USA). The detailed nutrient composition of the study products is shown in Table S7.

For a better taste of PT and for the blinding, 1.3 g vegetarian bouillon powder (Gemüse Bouillion, Knorr, Hamburg, Germany) was added to the lyophilised biomass/supernatant. The study population was divided into four groups receiving different study products. The first study product was the *Comp*, which consisted of daily 1.3 g of vegetarian bouillon powder solved in 150 mL plain water. The second study product *A* consisted of 2.3 g of lyophilised biomass A containing 312.1 mg n−3 FA (293.5 mg EPA+DHA) per day and additionally 1.3 g of vegetarian bouillon powder suspended in water (Table 3). The amount of PT was chosen based on the national n−3 PUFA/EPA + DHA recommendation of 250 to 300 mg per day. The third study product was based on β-G recommendations of yeast β-G by the EFSA, which is 600 mg per day [73]. Because Chrl is not yet a novel food, a total concentration of 500 mg Chrl was taken, which was 1.8 g daily of lyophilised Sup B biomass and 1.3 g vegetarian bouillon powder suspended in water (*SupB*). The fourth study product *A+SupB* consisted of 2.3 g of biomass A, 1.8g of SupB and 1.3 g of vegetarian bouillon powder suspended in water (Table 3). The study groups are named as the study products *Comp*, *A*, *SupB* and *A+SupB*.

All participants were given a diary to document their intake of the study products. The participants were instructed to suspend the study products freshly in a glass of water (around 150 mL) before usage and to take these products at breakfast time. To meet the compliance criteria, participants had to achieve 100% compliance, if the intake was forgotten for one day, the intake was extended by one day. The study was single-blinded; study participants were blinded for the study products. The randomization was generated using the Randlist software (datinf GmbH, Tübingen, Germany, available at randomisation.eu).

### 4.4. Blood Plasma, Serum and Faecal Measurements

Blood samples were collected in two ethylenediaminetetraacetic acids (EDTA)-coated tubes, and one serum tube. One EDTA- tube was used for blood count analysis (Sindelfingen laboratory GbR, Sindelfingen, Germany). The other one was centrifuged at 500 g for 7.5 min at 15 °C, followed by plasma separation. Plasma was stored at −80 °C until further analysis (FA, carotenoid, inflammatory markers, LBP). The RBC (underneath the centrifuged plasma) were washed with NaCl and centrifuged three times and stored at −80 °C. The serum tube was centrifuged for 15 min at 3000× g at 15 °C. 1 mL Serum was stored at −80 °C and the rest was used for quantification of gamma-glutamyl transferase (γ-GT), aspartate aminotransferase (AST), alanine transaminase (ALT), prealbumin, albumin, triacylglycerols (TAG), total cholesterol (Chol), high-density lipoprotein cholesterol (HDL), low-density lipoprotein cholesterol (LDL), haemoglobin, haematocrit, erythrocytes, leucocytes, platelet count, haemoglobin beta-N-1-deoxy fructosyl component of haemoglobin (HbA1c), insulin, HOMA-index, plasma glucose, thyroid-stimulating hormone = thyrotropin (TSH), C-reactive protein (CRP), uric acid, 25-hydroxy vitamin D (25(OH)D) (measured at Laborärzte Sindelfingen GbR, Sindelfingen, Germany). 

Stool samples were collected before the study visit (max. two days before) in two tubes and stored at −20 °C at home or transported directly to the laboratory. In our laboratory, the two tubes were stored at −80 °C until further analysis. One tube was used to measure the gut barrier marker zonulin and the other for SCFA analysis. 

### 4.5. Antioxidant Assays

The total phenolics content (TPC) was measured as was measured using Folin–Ciocalteu method as described previously for microalgae (compounds) by Neumann et al. [21]. It is expressed as gallic acid equivalents (GAE). The unit GAE (mg/g dry weight (DW)) describes how many milligrams of gallic acid are needed to achieve the same antioxidant effect as one gram of (biomass) sample. For conducting the TPC assay a sample solution with a defined biomass concentration was prepared. 20 mg freeze-dried biomass sample was suspended in 5 mL of dimethyl sulfoxide. 150 µL Folin–Ciocalteu reagent (diluted 1:10 in ddH_2_O) and 120 µL sodium carbonate solution (75 g/L) were mixed with 30 µL sample in a 96-well plate. The samples were then incubated and protected from light for 120 min at room temperature. Afterwards, the absorbance was measured at 765 nm using a plate reader (infinite M200 PRO, Tecan Group, Männedorf, Switzerland). Each sample was analysed in triplicate. As a blank, each sample was mixed with sodium carbonate solution and ddH_2_O, to obtain an individual blank for each sample. Using a calibration curve, made with gallic acid (10–50 mg/L), the total phenolic content of the defined sample solution was calculated as gallic acid equivalents (GAE in mg/L). Via the biomass concentration of the defined sample solution (see above), the total phenolic content per (biomass) dry weight (DW) was calculated (GAE in mg/g_DW_). As part of the antioxidative effect, the ability of substances to reduce substances by absorbing electrons was quantified by the ferric reducing antioxidant power assay (FRAP) and expressed in the form of FRAP values (ferric-reducing-antioxidant-power-values). The unit FRAP (mM/g_DW_) describes how many μM of iron (II) sulphate are needed to produce the same antioxidant effect as one gram of (biomass) sample. The FRAP assay was performed based on the method of Benzie and Strain [74] as described by Neumann et al. [21]. For conducting the assay a solution with a defined biomass concentration was prepared as already described for the TPC assay. FRAP reagent was prepared with 10 mL sodium acetate buffer (300 mM, pH 3.6), 1 mL TPTZ solution (10 mM in 40 mM HCl) and 1 mL of iron (III) chloride solution (20 mM) immediately before the experiment and stored at 37 °C until use. In a 96-well plate, 220 µL FRAP reagent was mixed with 10 µL of sample solution before incubation at 37 °C for 90 min. Absorbance was measured at 593 nm using a plate reader (infinite M200 PRO, Tecan Group). Each sample was analysed in triplicate. Individual blanks for each sample were prepared by mixing 10 µL of sample with 220 µL of ddH_2_O. To establish a calibration curve iron (II) sulphate was used (100–1000 µM) and the results are presented as FRAP values (iron (II) sulphate equivalents. 

### 4.6. Quantification of Plasma and Erythrocyte Fatty Acids, Carotenoids and Tocopherols

The same protocol was used for plasma and erythrocyte fatty acids quantification by gas chromatography with mass spectrometry (GC/MS) as described before [8]. The transesterification was performed as previously reported with slight modification [75]. In short, 2 μL 10,11-dichloro-undecanoic acid (DC-11:0) as internal standard and 2 mL methanol (Carl Roth, Karlsruhe, Germany) with 1% sulphuric acid for transesterification were added to 100 μL sample. During incubation at 80 °C for 1 h, the samples were sonicated three times for 5 min. Thereafter, samples were cooled down on ice, mixed with 0.5 mL demineralised water and 0.35 saturated NaCl solution and extracted with 2 mL n-hexane. Prior to measurement, 5 μL tetradecanoic acid- ethyl ester (14:0) was added as the second internal standard. Fatty acid methyl esters (FAMEs) were analysed by GC/MS on a 5890 series II/5972A system (Hewlett-Packard, Waldbronn, Germany). A commercial standard (Supelco, Taufkirchen, Germany) was used for identification based on mass spectra and retention times in full scan mode, quantification was carried out in selected ion monitoring mode [76]. Five saturated fatty acids (14:0, 16:0, 18:0, 20:0, 22:0) were measured by GC/MS. Unsaturated fatty acids were measured in form of the monounsaturated fatty acids (MUFAs) 14:1 n−5 (myristoleic acid), 16:1, 16:1 n−7 (palmitoleic acid), 17:1, 18:1 n−9 (oleic acid), 18:1 (isomer of oleic acid), 20:1 n−9 (gondoic acid) and the polyunsaturated fatty acids (PUFAs) 18:2 n−6 (LA), 20:2 n−6 (eicosadienoic acid), 18:3 n−6 (γ-linolenic acid), 18:3 n−3 (ALA), 20:3 n−6 (dihomogammalinolenic acid), 20:4 n−6 (AA), 20:5 n−3 (EPA), 22:5 n−3 (DPA), 22:6 n−3 (DHA). The n−6:n−3 ratio in plasma levels was calculated from the total area of the n−6 FA (18:2 n−6, 20:2 n−6, 18:3 n−6, 20:3 n−6, 20:4 n−6) divided by the total area of the n−3 FA (18:3 n−3, 20:5 n−3, 22:5 n−3, 22:6 n−3). For the AA/EPA ratio, percentual contributions of both fatty acids were determined and divided through each other. According to its definition, the Omega-3 index was determined by the percentual share of EPA and DHA in erythrocyte membranes relative to the sum of 26 fatty acids like in the method of Omegametrix (HS-Omega-3-Index^®^) [77].

Carotenoids and tocopherols were measured in plasma as described in detail previously. For FX, FXOH and A × A analysis, 100 µL of human plasma were mixed with 200 µL of ethanol/butanol (50/50 (*v*/*v*)) containing 5 mg butylated hydroxytoluene (BHT). After vigorous mixing and centrifugation (17,000× *g* and 4 °C, 10 min, Heraeus Fresco 17, Thermo Fischer Scientific, Waltham MA, USA) clear supernatants were injected into a Shimadzu HPLC system (Mc Kinley Scientific, New York, USA) equipped with an autosampler (15 °C), an UV detector (450 nm) and a C18 reversed-phase column (2.6 µm F5 100Å 150 × 4.6 mm, Kinetex, Phenomenex Ltd., Aschaffenburg, Germany) at 40 °C. A mixture of methanol/water (85/15 (*v*/*v*)) at 1.0 mL/min for 15 min was used as mobile phase [8]. 

For determination α/γ-tocopherol, lutein/zeaxanthin, lycopene, β-cryptoxanthin, α/β-carotene, and retinol, 40 µL of plasma were mixed with 200 µL of ethanol/butanol (50/50 (*v*/*v*)) containing 12 µL beta-apo-8′-carotenal-methyloxime/100 mL as internal standard. After vigorous mixing and centrifugation (17,000× *g* and 4 °C. 10 min; Heraeus Fresco 17, Thermo Fischer Scientific, Waltham, MA, USA), clear supernatants were injected into a Shimadzu HPLC system (Mc Kinley Scientific, New York, NY, USA) equipped with an autosampler (5 °C), and a ReproSil 80 ODS-2 column (3 µm, 250 × 4.6 mm) (Dr. A. Maisch GmbH, Ammerbuch-Entringen, Germany) at 40 °C. A mixture of acetonitrile/1.4-dioxane/methanol (82/15/3; *v*/*v*) containing 100 mmol/L ammonium acetate and 0.1% trimethylamine, at 1.5 mL/min for 20 min was used as mobile phase. Carotenoids were detected using an UV detector (450 nm). Retinol and tocopherols were detected using a fluorescence detector (Ex/Em at 325/ 470 nm for retinol and Ex/Em at 296/325nm for α/γ-tocopherol). carotenoids and tocopherols were quantified using authentic commercial standards and respective standard curves [78]. Quantification of FX [8], other carotenoids and tocopherols [79] in study products were performed as described before. 

### 4.7. Inflammatory Markers and Anti-Oxidative Stress Parameter

The inflammatory markers were performed using the Human IL-10 ELISA Kit (RAB0244), the Human IL-6 ELISA Kit (RAB0306; Millipore Sigma Aldrich, Saint Louis, USA) and Human TNF-α Immunoassay (HSTA00E; R&D Systems, Inc., Minneapolis, USA) according to the manufacturer’s protocol. For IL-10 and IL-6 plasma was used and TNF-α serum was used. For an oxidative stress marker, the xanthin oxidase fluorometric assay kit was performed following the manufacturer’s protocol (Item No. 10010895; Cayman).

### 4.8. Analysis of the Intestinal Permeability Marker Plasma LBP, Faecal Zonulin, and Faecal SCFA

The measurement of the faecal samples for zonulin measurement were diluted to the working concentration in sample buffer using stool sample tubes (K6998SAS; Immundiagnostik AG, Bensheim, Germany) and for LBP analysis, 10 μL of blood plasma was used and processed. Zonulin and LBP were measured using the enzyme-linked immunosorbent assay kit (K5600, KR6813, Immundiagnostik AG, Bensheim, Germany) following the manufacturer’s protocols. For SCFA analysis the same method was used as described before [8] by gas chromatography (Clarus 690, Perkin-Elmer, Waltham, MA, USA).

### 4.9. BIA, Muscle Function Test (5 STS, WOMAC, Gait Speed, Hand Grip Strength)

The BIA was done as a multi-frequency BIA according to Kyle et al. [80] (Data Input, Pöcking, Germany). The individual ASM and ASMI were calculated with the dietic pocket guide (www.dieteticpocketguide.com (accessed on 21 September 2022)) with the individual weight, gender, age, body height, BIA resistance (50 kH) and reactance (50 kH). 

For muscle function, the 5-STS was conducted as previously described by Jones et al. [81]. Using the WOMAC questionnaire, the activity levels of the study participants were assessed at weeks zero and eight. WOMAC was developed for patients with osteoarthritis and registers signs of physical disability and relevant changes in health status because of treatment intervention [82]. This questionnaire consists of three scales: pain, stiffness, and function. The higher the WOMAC value, the higher the pain, stiffness, and functional limitations. The maximum score which can be achieved is 96 (maximum score for pain is 20, 8 for stiffness and 68 for functional limitations). Gait speed in elderly was assessed in meters per second measured as a four-meter usual walking speed test two times. The hand grip strength was measured on each side two times (Hydraulic hand dynamometer, Jamar).

### 4.10. Diet Evaluation

Food diary of four weeks was evaluated with EPISpro 2016 (Software, Willstätt-Legelshurst, Germany). The dietary pattern was documented by the validated German Food Frequency Questionnaire (FFQ) [83].

### 4.11. Statistical Analyses

All parameters were tested for normal distribution using the Kolmogorov–Smirnov test. Normally distributed data, one-way ANOVA was used to compare statistically significant differences (*p* < 0.05) between microalga diet groups and the *Comp*. Variances were tested with the Brown–Forsythe test. Tukey’s multiple comparison post hoc test was used for equal variances, and Dunnett’s T3 multiple comparisons test was used for unequal variances. Within one group a t-test was performed between weeks two and four or study entry and week four. The study product treatment effect was measured as differences between the values of weeks 4 and 2 (Delta, Δ). This change was calculated for each subject. The four groups were compared with each other with a one-way ANOVA and a t-test respectively to the *Comp* group. For the mortality and body composition data, the value after intervention (4) minus the study start value (0) was calculated. Correlation analyses were performed with two-tailed Spearman-rank correlation. All statistical analyses were performed using GraphPad Prism version 9.4.1 (GraphPad Software, San Diego, CA, USA) and IMB SPSS statistics 25 (IBM Corp., Armonk, NY, USA).

## 5. Conclusions

The study demonstrates anti-inflammatory effects and potential antioxidant effects, as well as possible preservation of functional ability after ingestion of the whole microalgae and the β-G rich supernatant in the elderly. In addition, the present results provide the first evidence of the safe use of the β-G-rich microalgae supernatant over a two-week period and reconfirm the safe ingestion of whole PT in humans. Supplementation with PT could be a source of functional compounds that contribute to healthy aging from a nutritional perspective and, could be a candidate for anti-aging effects as a supplement. Higher doses of PT with higher amounts of functional compounds are warranted for future interventions.

## Figures and Tables

**Table 1:** Characteristics of the 19 study participants.

	*All* (*n* = 19)	*Comp* (*n* = 5)	*A* (*n* = 5)	*SupB* (*n* = 5)	*A+SupB* (*n* = 4)
Female/Male [n]	12/7	2/3	4/1	3/2	3/1
Anthropometry					
Age [years]	67.7 ± 6.5	67.4 ± 7.9	65.4 ± 4.7	71.4 ± 5.7	67 ± 9.2
BMI [kg/m²]	24.6 ± 3.1	26.9 ± 2.5	25.5 ± 1.8	22.3 ± 2.9	23.3 ± 3.7
Waist circumference [cm]	90.2 ± 11.6	100.5 ± 8.7	93.6 ± 4.5	81.2 ± 11.4	84.5 ± 11.2
Blood biomarkers					
Cholesterol (chol.) [mg/dl]	224.6 ± 50.0	248 ± 43.5	198 ± 44.7	219 ± 63.2	235 ± 46.5
Triglycerides [mg/dl]	99.9 ± 60.0	141 ± 102.1	83.2 ± 35.7	74 ± 26.7	101.8 ± 26.5
HDL-chol. [mg/dl]	71.1 ± 18.4	71.8 ± 24.2	64 ± 14	72.2 ± 12	77.8 ± 25.7
LDL-chol. [mg/dl]	126.4 ± 46.8	152.2 ± 24.1	120.2 ± 40.9	129.8 ± 51.2	129.7 ± 11.7
Insulin [µE/mL]	7.8 ± 4.1	10.8 ± 6.6	7.6 ± 2.8	5.9 ± 2.6	6.7 ± 1.5
HOMA-Index	1.9 ± 1.7	3.1 ± 3	1.7 ± 0.6	1.4 ± 0.6	1.5 ± 0.2

Values are expressed as mean ± standard deviation (SD). Abbreviations: diets see Figure 2; *Comp*, Comparator; BMI, body mass index; chol, cholesterol; HDL, high-density lipoprotein; LDL, low-density lipoprotein. Statistics: Comparison of groups revealed no difference for all parameters listed in the table (*p* >0.05, ANOVA) except the waist circumference (*p* < 0.001).

**Table 2:** Adverse effects during the two weeks intervention within the four study groups.

Side Effects	*Comp* (*n* = 5) Diary/Protocol			*A* (*n* = 5) Diary/Protocol			*SupB* (*n* = 5) Diary/Protocol			*A+SupB* (*n* = 4) Diary/Protocol		
	Min.	Mild	Sev.	Min.	Mild	Sev.	Min.	Mild	Sev.	Min.	Mild	Sev.
Abdominal rumbling	-	-	-	2│1_1_	-	-	-	-	-	-	-	-
Flatulence	-	1│0_2_	-	-	-	-	-	1│1_3_	-	-	1│0	-
Stomach pain	-	-	-	-	-	-	-	0│1	-	-	-	-
Diarrhoea	-	1│1	-	-	-	-	-	1│0	-	-	1│0	-
Discoloration of the stool	-	-	-	-	-	-	-	-	-	1│0	-	-
Decreased frequency of bowel movements	-	-	-	-	-	-	-	-	-	1│0	-	-
Belching (at least 1×)	-	-	-	-	-	-	-	-	-	1│1	-	-
Headache	-	-	-	-	-	-	-	-	-	1│0	1│0	-
Increased blood pressure	-	1│0	-	-	-	-	-	-	-	-	-	-
Increased urge to urinate	-	-	-	-	-	-	-	-	-	1│0	-	-
Nausea	-	-	-	1│1	-	-	-	-	-	-	-	-

Adverse effects were documented in a diary by the participants and recorded upon questionnaire by the study personnel during the visits. Values from completers are expressed as absolute numbers. Minimal, transient symptoms with no impairment of the patient’s daily activities; Mild, consistent symptoms with moderate impairment of the patient’s daily activities; Severe, significant impairment of the patient’s daily activities. Abbreviations: diets see Figure 2; *Comp*, Comparator; Min., minimal; Sev., severe; *_1_*_,_ the side effect was documented by two participants in a diary and one time during the protocol; *_2_*, the side effect was documented one time in a diary but not during the protocol; *_3_*, the side effect was documented one time in a diary and one time during the protocol.

**Table 3:** Daily nutrient intake of the participants through the study products.

Daily Nutrient Intake	*Comp*	*A*	*SupB*	*A+SupB*
Protein g/day	0.007	0.81	0.05	0.85
β-1,3-glucan g/day	0	0.17	0.54	0.71
FAs [mg/day]	13.02	678.3	22.9	688.2
n−3 FA	0.34	312.51	1.79	313.97
n−6 FA	5.19	63.56	5.57	63.94
SFA	3.69	90.85	7.35	94.51
MUFA	3.80	104.38	7.70	108.28
PUFA	5.53	483.07	7.85	485.38
n−6: n−3 ratio	20.00	20.43	20.47	20.90
AA/EPA ratio	0.00	0.22	0.00	0.22
EPA+DHA	0.03	293.65	1.20	294.82
EPA	0.02	288.52	1.44	289.94
DHA	0.01	5.13	0.06	5.19
Carotenoids [mg/day]				
Fucoxanthin	0.00	21.39	0.22	21.61
β-carotene	0.01	0.29	0.01	0.30
α-carotene	0.00	0.00	0.00	0.00
Lycopene	0.00	0.21	0.01	0.21
Vitamine E [mg/day]				
α-Tocopherol	0.00	0.10	0.03	0.13
β-Tocopherol	0.00	0.00	0.00	0.00
γ-Tocopherol	0.00	0.01	0.00	0.01

Abbreviations: diets see Figure 2; FA, fatty acids; n−3 FA, omega-3 fatty acids; n−6 FA, omega-6 fatty acids; SFA, saturated fatty acids; MUFA, monounsaturated fatty acids; PUFA, polyunsaturated fatty acids; n−6: n−3, n−6 FA to n−3 FA ratio; AA/EPA, arachidonic- and eicosapentaenoic acid ratio; EPA, eicosapentaenoic acid; DHA, docosahexaeonic acid.

## Data Availability

Not applicable.

## Supplementary Materials

The following supporting information can be downloaded at: https://www.mdpi.com/article/10.3390/md20110716/s1, Table S1: Food Frequency Questionnaire at the study start; Table S2: Laboratory parameter and inflammatory markers; Table S3: Mobility markers and body composition at baseline and the study change; Table S4: Plasma fatty acid blood composition at different time-points; Table S5: Carotenoid plasma levels at the study start, after two- weeks and four weeks of intervention; Table S6: The concentration of short-chain fatty acids (SCFA) per dry mass in faecal samples at week 2 and week 4 after the intervention within the four study groups; Table S7: Nutrient composition of *Comp, A, SupB* and *A+SupB* diets used in the study.
