# Supplementary material for: Potentially Beneficial Effects on Healthy Aging by Supplementation of the EPA-Rich Microalgae Phaeodactylum tricornutum or Its Supernatant—A Randomized Controlled Pilot Trial in Elderly Individuals
Source: Mar Drugs. 2022 Nov 15;20(11):716. doi: 10.3390/md20110716 (PMC9694444; doi:10.3390/md20110716)
Supplement: Supplementary file 1 [file marinedrugs-20-00716-s001.zip › marinedrugs-1971977-supplementary.pdf]

marine drugs

MDPI

Potentially Beneficial Effects on Healthy Aging by Supplementation of the EPA-Rich Microalgae *Phaeodactylum tricornutum* or Its Supernatant—A Randomized Controlled Pilot Trial in Elderly Individuals

Authors: Lena Stiefvatter, Konstantin Frick, Katja Lehnert, Walter Vetter, Alexander Montoya-Arroyo, Jan Frank, Ulrike Schmid-Staiger, and Stephan C. Bischoff

Supplementary Materials

Table S1. Food Frequency Questionnaire at the study start

	All			*Comp*			*A*			*SupB*			*A+SupB*		
	0			*0*			*0*			*0*			*0*		
	**(*n*= 19)**			**(*n*= 5)**			**(*n*= 5)**			**(*n*= 5)**			**(*n*= 4)**		
**FFQ**															
Energy [kcal]	1523	±	348	1414	±	311	1668	±	363	1498	±	437	1510	±	390
Protein [g]	64.8	±	20.4	53.4	±	9.8	84.4	±	23.3	61.6	±	13.2	58.7	±	24.9
Fat [g]	49.8	±	14.6	46.4	±	6.4	47.8	±	11.3	49.8	±	17.8	56.7	±	24.6
PUFA [g]	7.6	±	3.2	6.0	±	1.2	7.8	±	2.7	7.8	±	3.0	8.9	±	5.9
Carbohydrates[g]	179.7	±	53.9	157.5	±	54.0	208.7	±	77.1	178.1	±	56.4	173.3	±	9.3
Fibre [g]	19.2	±	7.3	14.3	±	4.2	23.6	±	10.6	20.6	±	7.7	18	±	0.4
Carotenoid[mg]	3.93	±	2.1	3.0	±	1.0	4.1	±	2.0	4.0	±	2.3	4.3	±	2.7
Vitamin A [µg]	974.6	±	370	849.7	±	271	1014.6	±	390	984.1	±	390	1069.0	±	386
Vitamin E [µg]	7.3	±	2.4	5.2	±	1.0	8.0	±	2.6	8.0	±	2.4	8.1	±	2.2

Values are expressed as mean ± standard deviation (SD) at the study start (time-point 0). Abbreviations: FFQ, Food Frequency Questionnaire; PUFA, polyunsaturated fatty acid.

Table S2. Laboratory parameter and inflammatory markers

Blood biomarkers	*All* 0 (*n*= 19)	*All* 2 (*n*= 19)	*Comp* 2 (*n*= 5)	*Comp* 4 (*n*= 5)	Δ *Comp*	*A* 2 (*n*= 5)	*A* 4 (*n*= 5)	Δ *A*	*SupB* 2 (*n*= 5)	*SupB* 4 (n= 5)	Δ *SupB*	**A+SupB** 2 (n= 4)	**A+SupB** 4 (n= 4)	Δ *A+SupB*
**Plasma glucose (%)**	95.58±16.6	94.63±15.9	104.6 ± 27.1	96.4 ± 17.1	-8.20±11.3	91.8 ± 13.2	85.8 ± 5.8#	-6.00±7.9	92.8 ± 8.6	89.2 ± 7.6	-3.60±5.3	88.0 ± 6.8	86.25 ± 3.3	-1.75±4.0
**HbA1c [%]**	5.50±0.5	5.47±0.5	5.68 ± 0.9	5.66 ± 0.8	-0.02±0.5	5.48 ± 0.4	5.54 ± 0.3	1.16±2.3	5.4 ± 0.1	5.44 ± 0.2	1.12±2.3	5.3 ± 0.2	5.25 ± 0.2	-0.03±0.0
**Cholesterol [mg/dl]**	4.92±1.3	218.26±44.0	223.2 ± 46.8	223.6 ± 50.9	0.40±31.6	207.4 ± 29.5	209.4 ± 44.1	2.00±17.3	209.8 ± 59.6	206.2 ± 59.1(*)	-3.60±2.9	236.3 ± 51.8	238.5 ± 57.3	2.25±8.0
**Triglycerides [mg/dl]**	224.58±50.0	100.89±56.5	130.2 ± 103	115.6 ± 46.1	-14.60±52.5	101.8 ± 42.6	83.6 ± 21.9	-18.20±27.7	74.6 ± 22.2	65.8 ± 17.1*(#)	-8.80±5.9	96 ± 17.6	117.5 ± 67.5	21.50±44.8
**HDL-cholesterol [mg/dl]**	99.89±60.1	69.95±17.7	67.4 ± 24.5	70.6 ± 28.9	3.20±9.6	65.6 ± 12.3	64.2 ± 9.8	-1.40±4.4	71.4 ± 13.6	69.8 ± 13.0*	-1.60±1.0	76.75 ± 24.9	73.75 ± 23.8	-3.00±2.2
**LDL-cholesterol [mg/dl]**	71.11±18.4	130.42±31.3	134.8 ± 27.7	139.6 ± 30.4	4.80±24.8	127.4 ± 33.3	135.6 ± 45.1	8.20±13.3	121.6 ± 44.9	128 ± 47.1*	6.40±4.2	139.8 ± 27.0	144.5 ± 37.5	4.75±9.7
**LDL/HDL ratio**	2.02±0.8	2.01±0.7	2.22 ± 0.9	2.28 ± 1.0	0.06±0.3	2.04 ± 0.7	2.16 ± 0.8	0.12±0.2	1.76 ± 0.7	1.86 ± 0.8*	0.10±0.1	2.03 ± 0.9	2.1 ± 0.9	0.08±0.1
**TSH [mU/l]**	2.12±2.9	1.86±1.4	2.142± 2.3	1.14 ± 0.5	-1.01±2.1	1.46± 0.9	1.50 ± 1.2	-0.13±0.2	1.52 ± 0.8	1.56± 0.8	0.05±0.2	2.22 ± 1.2	2.16 ± 1.04	-0.07±0.3
**Insulin [µE/ml]**	7.82±4.1	7.64±2.2	9.06 ± 2.6	7.66 ± 1.0	-1.40±1.7	8.18 ± 1.1	6.28 ± 2.0*	-1.90±1.0	6.26 ± 2.2	5.96 ± 2.0	-0.30±1.5	6.93 ± 2.5	6.83 ± 3.1	-0.10±0.6
**HOMA-Index**	1.95±1.7	1.78±0.6	2.34 ± 0.8	1.78 ± 0.5	-0.52±0.3	1.82 ± 0.3	1.28 ± 0.3*	-0.54±0.3	1.46 ± 0.6	1.32 ± 0.5	-0.14±0.4	1.45 ± 0.4	1.425 ± 0.6	-0.03±0.2

Values are expressed as mean ± SD. Δ is the change from week 4 to week 2 as treatment effect. Abbreviations: diets see Figure 2; HbA1c, haemoglobin beta-N-1-deoxy fructosyl component of haemoglobin; HDL, high-density lipoprotein; LDL, low-density lipoprotein; TSH; thyroid-stimulating hormone = thyrotropin; HOMA index, Homeostasis model assessment for insulin resistance. Statistics: * indicate difference within one group between week two (2) and week four (4) (paired t-test), # indicate difference to week 0 (ANOVA with Tukey post hoc test. (*, #) $p < 0.1$

Table S3. Mobility markers and body composition at study start and the study change

Parameters	*0*	Δ			
	All **(*n*= 19)**	***Comp*** **(*n*= 5)**	***A*** **(*n*= 5)**	***SupB*** **(*n*= 5)**	***A+SupB*** **(*n*= 4)**
WOMAC	9.2 ± 10.4	4.3 ± 8.7	-1.25 ± 1.9	4.5 ± 8.4	1.0 ± 2.0
Gait speed [m/s]	4.4 ± 0.7	0.2 ± 0.4	-0.2 ± 0.3	-0.1 ± 0.4	-0.5 ± 0.6(*)
5-STS	10.4 ± 3.1	0.7 ± 0.3	-0.7 ± 1.2*	0.1 ± 0.5(*)	1.0 ± 0.8
Handgrip strength [kg]	30.4 ± 11.2	-2.6 ± 5.8	1.0 ± 2.1	-1.5 ± 2	-0.7 ± 1.6
Body fat mass [%]	26.2 ± 7.3	-0.2 ± 0.6	-0.2 ± 0.4	-0.8 ± 0.5	-1.2 ± 1.1
Body cell mass [kg]	24.2 ± 9.3	0.5 ± 0.8	0.5 ± 0.6	0.1 ± 0.8	0.3 ± 0.5
Lean body mass [kg]	51.1 ± 10.6	1.2 ± 1.1	-0.5 ± 1.3(*)	-0.3 ± 0.6*	-0.2 ± 0.7(*)
ASM [kg]	18.17 ± 4.3	-0.8 ± 1.3	-0.3 ± 0.9	-1.0 ± 1.5	-0.4 ± 0.9
ASMI [kg/m²]	6.3 ± 0.8	-0.2 ± 0.2	-0.1 ± 0.3	-0.4 ± 0.5	-0.1 ± 0.2

The body cell mass is defined as the muscle and organ cell mass. The lean body mass is the fat free mass, the body cell mass and the extracellular mass (interstitium, bone, connective tissue). Values are expressed as mean ± SD from 19 participants at study start (0) (*All* n= 19; *Comp*, *A*, *SupB* each

n= 5; *A+SupB* *n*= 4) and Δ is the change from week 4 to week 2 as treatment effect. Statistics: * treatment effect indicate difference to the *Comp* group (t-test) (*) $p < 0.1$, * $p < 0.05$. Abbreviations: diets see Figure 2; WOMAC, Western Ontario and McMaster Universities Arthritis Index; ASM; Appendicular skeletal muscle mass; ASMI, Appendicular skeletal muscle mass index.

Table S4. Plasma fatty acid blood composition at different time-points

**FA plasma [%]**	***All* 0 (*n*= 19)**	***All* 2 (*n*= 19)**	***Comp* 2 (*n*= 5)**	***Comp* 4 (*n*= 5)**	**Δ *Comp***	***A* 2 (*n*= 5)**	***A* 4 (*n*= 5)**	**Δ *A***	***SupB* 2 (*n*= 5)**	***SupB* 4 (*n*= 5)**	**Δ *SupB***	***A+SupB* 2 (*n*= 4)**	***A+SupB* 4 (*n*= 4)**	**Δ *A+SupB***
MUFA	26.5±3.0	26.07±3.2	27.11±4.7	27.65±4.4	0.54±1.6	27.16±3.9	25.71±2.1	-1.46±3.0	25.51±1.7	24.31±3.1	-1.19±1.6	25.37±2.4	26.38±2.8	1.01±3.1
PUFA	43.53±4.5	43.51±4.4	40.58±6.3	42.51±4.9	1.93±3.0	42.85±4.2	45.30±3.4	2.45±3.9	45.93±2.2	47.01±3.7	1.08±2.0	44.99±2.5	43.14±5.5	-1.84±3.2
n-3	6.8±2.2	5.8±1.3$	4.49±1.0	5.40±1.0**	0.91±0.4	6.24±1.1	6.45±1.2	0.21±0.6	6.47±1.5	6.95±1.6	0.48±1.0	5.81±0.7	6.49±0.3(*)	0.67±0.5
18:3 n-3	0.93±0.4	1.05±0.4($)	0.83±0.3	0.99±0.2(*)	0.16±0.1	0.92±0.4	0.82±0.4(*)	-0.10±0.1^{++}	1.33±0.5(#)	1.47±0.4#	0.14±0.3	1.14±0.2	1.17±0.4 (*)	0.03±0.4
EPA, 20:5 n-3	1.80±0.9	1.24±0.5$$	0.96±0.3#	1.27±0.6(*)	0.31±0.3	1.38±0.6	1.65±0.6(*)	0.27±0.3	1.49±0.7	2.04±0.9(*)	0.55±0.6	1.10±2.0	1.70±0.3**	0.32±0.6
22:5 n-3	0.77±0.2	0.7±0.2($)	0.60±0.1#	0.76±0.2(*)	0.16±0.1	0.78±0.1	0.83±0.1	0.05±0.1	0.79±0.2	0.82±02	0.05±0.2	0.63±0.1	0.69±0.2	0.06±0.1
DHA, 22:6 n-3	3.3±1.2	2.76±0.7$$	2.10±0.49#	2.38±0.6(#)	0.28±0.3	3.16±0.9	3.15±0.8	-0.01±0.2	2.87±0.6	2.62±0.4(*)	-0.25±0.2+	2.95±0.4	2.88±0.3	-0.07±0.3
EPA+DHA	5.0±2.0	4.0±1.1$$	3.06±0.7#	3.65±0.8*	0.59±0.3	4.54±1.3	4.80±1.3	0.26±0.5	4.36±1.1	4.66±1.1	0.30±0.7	4.10±0.5	4.30±0.5	0.25±0.8
n-6	36.8±4.3	37.8±3.9($)	36.09±5.9	37.10±4.2	1.01±3.0	36.61±3.7	38.85±4.1	2.24±3.8	39.47±2.5	40.06±3.6	0.59±1.6	39.17±2.2	36.65±5.2	-2.52±3.1
20:4 n-6	8.38±2.2	8.53±2.2	8.09±1.5	8.43±1.0	0.34±0.9	9.66±3.7	10.18±2.8	0.52±1.0	8.11±1.1	8.47±0.9*	0.36±0.2	8.20±2.0	7.60±2.3	-0.60±0.7
n-6:n-3	6.03±2.1	7.26±1.9$	8.32±2.1#	6.97±0.9 (*)	-1.35±1.4	7.42±2.4	6.25±1.6	0.28±0.9($^{+}$)	6.43±1.7	6.12±2.0	-0.31±0.9	6.79±0.1	5.64±0.6 *	-1.15±0.6
AA/EPA	5.72±2.6	7.53±2.3$$	8.93±2.5#	7.60±2.7	-1.32±3.3	7.42±2.4	6.40±1.3	-1.02±2.0	6.39±2.9	5.30±3.4(*)	-1.10±1.1	7.38±0.7	4.54±0.8**	-0.44±1.4

Values are expressed as mean±SD. Δ is the change from week 4 to week 2 as treatment effect. Abbreviations: FA, fatty acids; n-3 FA, omega-3 fatty acids; n-6 FA, omega-6 fatty acids; SFA, saturated fatty acids; MUFA, monounsaturated fatty acids; PUFA, polyunsaturated fatty acids; n-6: n-3, n-6 FA to n-3 FA ratio; AA/EPA, arachidonic- and eicosapentaenoic acid ratio; EPA, eicosapentaenoic acid; DHA, docosahexaeonic acid. Statistics: $ indicates difference between study start (week 0) and after the wash out (week 2). * indicate difference within one group between week two (2) and week four (4) (paired t-test), # indicates difference to All time-point 0 (ANOVA with Tukey post hoc test or Dunnett´s). $^{+}$ indicates difference to Δ *Comp*. ($)/(*)/($^{\#}$)/(+) $p < 0.1$, */$^{\#}$ $p < 0.05$, $$/$^{++}$/** $p < 0.01$.

Table S5. Carotenoid plasma levels at the study start, after two- weeks and four weeks of intervention

Parameter [μM]	*All* 0 (*n*= 19)	*Comp* 2 (*n*= 5)	*Comp* 4 (*n*= 5)	*A* 2 (*n*= 5)	*A* 4 (*n*= 5)	*SupB* 2 (*n*= 5)	*SupB* 4 (*n*= 5)	*A+SupB* 2 (*n*= 4)	*A+SupB* 4 (*n*= 4)
Lutein	0.21 ± 0.06	0.26 ± 0.09	0.25 ± 0.12	0.17 ± 0.04	0.14 ± 0.08	0.18 ± 0.08	0.23 ± 0.13	0.2 ± 0.06	0.16 ± 0.07
β-Cryptoxanthin	0.13 ± 0.08	0.15 ± 0.13	0.11 ± 0.06	0.11 ± 0.06	0.1 ± 0.07	0.08 ± 0.03	0.1 ± 0.02*	0.11 ± 0.04	0.13 ± 0.01
Lycopene	0.53 ± 0.25	0.51 ± 0.23	0.42 ± 0.15	0.61 ± 0.2	0.57 ± 0.26	0.56 ± 0.43	0.47 ± 0.21	0.65 ± 0.28	0.62 ± 0.26
α-carotene	0.16 ± 0.14	0.19 ± 0.11	0.2 ± 0.17	0.18 ± 0.11	0.13 ± 0.08	0.21 ± 0.21	0.22 ± 0.1	0.10 ± 0.07#	0.13 ± 0.08*
Retinol	0.95 ± 0.33	0.93 ± 0.31	1.12 ± 0.23	0.94 ± 0.2	0.87 ± 0.15	0.73 ± 0.35	0.82 ± 0.1	0.72 ± 0.17#	0.81 ± 0.2
γ-Tocopherol	0.16 ± 0.06	0.18 ± 0.01	0.19 ± 0.04	0.16 ± 0.05	0.14 ± 0.04	0.15 ± 0.03	0.15 ± 0.02	0.15 ± 0.03	0.12 ± 0.01(*)
α-Tocopherol	23.63 ± 6.16	25.4 ± 8.03	24.55 ± 1.62	22.19 ± 4.35	21.47 ± 3.87	19.69 ± 7.44###	20.54 ± 5##	20.4 ± 1.31	21.02 ± 2.46

Values are expressed in μM as mean± *SD* (n= 19). Statistics: * indicate differences within one group between week two (2), after the wash-out and week four after two weeks intervention (4). # Indicates the difference between week 4 of different groups. */# $p < 0.05$, ## $p < 0.01$, ### $p < 0.001$. Abbreviations: diets see Figure 2.

Table S6. The concentration of short-chain fatty acids (SCFA) per dry mass in fecal samples at week 2 and week 4 after the intervention within the four study groups

**Fecal SCFA** [μmol/g] DM	*Comp* 2 (*n*= 5)	*Comp* 4 (*n*= 5)	*A* 2 (*n*= 5)	*A* 4 (*n*= 5)	*SupB* 2 (*n*= 5)	*SupB* 4 (*n*= 5)	*A+SupB* 2 (*n*= 5)	*A+SupB* 4 (*n*= 4)
Acetat	316.1± 133.3	323.0± 159.9	191.3± 82.6	205.4± 140.2	218.6± 105.7	258.8± 210.5	158.1± 42.8	128.4± 31.0
Propionat	100.5± 86.4	66.2± 32.5	34.9± 23.5	34.3± 28.2	45.3± 15.9	44.1± 22.3	35.4 ± 15.9	29.4 ± 8.7
Butyrat	46.6± 23.7	67.4± 38.8	30.7± 21.1	44.5± 41.9	39.0± 22.3	56.8± 55.5	25.4± 14.9	18.8± 7.3
Iso-butyric acid	46.6± 23.9	67.4± 38.8	30.7±21.1	44.5±41.9	39.0±22.3	56.8±55.5	25.4±14.9	18.8±7.3
Iso-valeric acid	6.7± 3.0	5.6±2.3	6.3±4.3	5.6±3.0	8.3± 2.4	6.6± 1.8	8.9± 1.7	7.7±1.9
Valeric acid	6.4± 3.0	6.8±3.1	4.1±3.0	4.0±3.1	6.0±2.6	5.0±2.3	5.2±1.8	4.2±1.4
Iso-caproic acid	0.6±0.2	0.4±0.1*	0.6±0.3	0.4±0.3	0.6±0.2	0.5±0.3	0.6±0.3	0.4±0.3
Hexanoic acid	2.1±1.7	4.3±2.8	2.8±3.1	3.2±2.6	3.9±3.9	4.1±4.5	1.9±1.2	1.4±0.6
Heptanoic acid	0.7±0.6	0.5±0.3	0.4±0.2	0.3±0.1	0.4±0.2	0.6±0.4	0.6±0.4	0.3± 0.2(*)

Values are expressed as mean± *SD*. Abbreviation: DM, dry mass. Statistics: * indicate difference within one group between week two (2), after the wash-out and week four after two weeks intervention (4) (paired t-test),. (*)/ $p < 0.1$, * $p < 0.05$.

Table S7. Nutrient composition of *Comp, A, SupB* and *A+SupB* diets used in the study

**Fatty acids [mg/g]**	***Comp***			***A****			***SupB****			***A+SupB****		
14:0	0.06	±	0.01	11.7	±	1.08	0.56	±	0.02	12.16	±	1.10
15:0	0.01	±	0.00	0.9	±	0.11	0.03	±	0.00	0.94	±	0.11
16:0	2.40	±	0.06	23.9	±	1.87	3.79	±	0.08	25.34	±	1.88
17:0	0.02	±	0.00	0.5	±	0.06	0.02	±	0.00	0.46	±	0.06
18:0	0.32	±	0.02	0.8	±	0.06	0.37	±	0.02	0.81	±	0.06
22:0	0.02	±	0.00	0.1	±	0.00	0.02	±	0.00	0.10	±	0.00
24:0	0.02	±	0.00	2.9	±	0.01	0.08	±	0.01	2.94	±	0.01
16:1	0.01	±	0.01	1.3	±	0.25	0.03	±	0.01	1.34	±	0.25
16:1 n-7	0.03	±	0.01	39.1	±	0.16	1.86	±	0.06	40.97	±	0.21
18:1 n-9 c	2.79	±	0.03	4.8	±	3.59	3.07	±	0.05	5.06	±	3.61
18:1	0.08	±	0.00	1.0	±	0.31	0.11	±	0.00	1.06	±	0.31
24:1 n-9	0.00	±	0.00	0.4	±	0.12	0.01	±	0.00	0.40	±	0.12
16:2	0.00	±	0.00	2.0	±	0.21	0.01	±	0.00	2.01	±	0.22
16:2 n-6	0.00	±	0.00	18.2	±	0.12	0.08	±	0.01	18.31	±	0.13
16:3	0.00	±	0.00	22.9	±	0.02	0.13	±	0.01	23.06	±	0.03
17:2	0.00	±	0.00	0.6	±	0.18	0.00	±	0.00	0.57	±	0.18
16:4	0.00	±	0.00	2.2	±	1.71	0.05	±	0.01	2.22	±	1.71
18:2 n-6	3.99	±	0.02	12.4	±	2.46	4.17	±	0.03	12.63	±	2.48
18:3 n-6	0.00	±	0.00	1.7	±	0.06	0.03	±	0.00	1.70	±	0.06
18:3 n-3	0.24	±	0.01	1.7	±	0.22	0.26	±	0.01	1.70	±	0.23
18:4 n-?	0.00	±	0.00	0.6	±	0.76	0.00	±	0.00	0.62	±	0.76
18:4 n-3	0.00	±	0.00	0.7	±	0.15	0.00	±	0.01	0.72	±	0.16
20:3 n-6	0.00	±	0.00	3.0	±	0.12	0.00	±	0.00	3.00	±	0.12
20:3 n-3	0.00	±	0.00	0.0	±	0.11	0.12	±	0.01	0.12	±	0.12
20:4 n-6	0.00	±	0.00	12.2	±	0.10	0.00	±	0.00	12.25	±	0.10
PUFA n-3	0.00	±	0.00	2.9	±	0.17	0.02	±	0.00	2.92	±	0.17
PUFA n-3	0.00	±	0.00	0.7	±	0.26	0.00	±	0.00	0.66	±	0.26
20:5 n-3	0.02	±	0.00	125.5	±	0.00	0.63	±	0.07	126.07	±	0.07
22:5 n-3	0.00	±	0.00	2.3	±	1.04	0.00	±	0.00	2.34	±	1.04
22:6 n-3	0.00	±	0.01	2.2	±	0.84	0.04	±	0.01	2.26	±	0.85
**Mean**	**10.0**	**±**	**0.04**	**299.3**	**±**	**2.08**	**15.5**	**±**	**0.04**	**304.8**	**±**	**1.1**
∑ n-3 PUFA	0.26	±	0.02	135.99	±	2.79	1.07	±	0.11	136.80	±	2.89
∑ n-6 PUFA	3.99	±	0.02	29.37	±	2.74	4.21	±	0.04	29.58	±	2.76
∑ SFA	2.84	±	0.09	40.74	±	3.19	4.87	±	0.13	42.77	±	3.22
∑MUFA	2.92	±	0.05	46.65	±	4.42	5.09	±	0.12	48.82	±	4.50
∑PUFA	4.25	±	0.03	211.88	±	8.54	5.54	±	0.18	213.17	±	8.68
n-6:n-3 ratio	15.38	±	0.95	0.22	±	0.98	3.94	±	0.34	0.22	±	0.96
AA/EPA ratio	0.00	±	0.00	0.10	±	39.29	0.00	±	0.00	0.10	±	1.49
EPA+DHA	0.02	±	0.01	127.68	±	0.84	0.67	±	0.08	128.33	±	0.91
β-Glucan [mg/g]	0.0	±	0	0.1	±	0	0.3	±	0	0.38	±	0
**Carotenoids [mg/Kg]**												
Fucoxanthin	0	±	0	9298.1	±	4456.9	123.7	±	0	9421.8	±	4456.9
β-carotene	5.0	±	0.13	129.7	±	32.89	10.01	±	0.93	134.79	±	33.69
α-carotene	3.49		0.11	3.49		0.11	3.5		0.13	3.5		0.13

Lycopene	4.29	±	0.14	94	±	13.06	7.82	±	0.5	97.53	±	13.42
Lutein	1.67	±	0.05	1.67	±	0.05	1.67	±	0.05	1.67	±	0.05
Zeaxanthin	0	±	0	1.64	±	0.51	0	±	0	1.64	±	0.51
β-Cryptoxanthin	0.06	±	0.01	1.1	±	0.15	0.12	±	0.02	1.16	±	0.16
**Tocopherol [mg/Kg]**												
α-Tocopherol	7.54	±	0.67	52.12	±	3.48	23.99	±	1.72	68.57	±	4.53
β-Tocopherol	0.23	±	0.03	0.23	±	0.03	0.23	±	0.03	0.23	±	0.03
γ-Tocopherol	3.79	±	0.12	4.44	±	0.28	3.79	±	0.12	4.44	±	0.28
δ-Tocopherol	0.13	±	0.01	0.13	±	0.01	0.13	±	0.01	0.13	±	0.01
α-Tocotrienol	0	±	0	0	±	0	0	±	0	0	±	0
β-Tocotrienol	0	±	0	0	±	0	0	±	0	0	±	0
γ-Tocotrienol	0	±	0	0.53	±	0.14	0	±	0	0.53	±	0.14

*with the vegetable bouillon powder
